# B3GNT8-mediated glycosylation maintains intestinal homeostasis and protects against colitis

**DOI:** 10.1016/j.jbc.2025.111014

**Published:** 2025-12-10

**Authors:** Haoyun Mao, Yi Cao, Ying Lu, Shicheng Peng, Bo Wu, Ying Wang, Yongtao Xiao

**Affiliations:** 1 Division of Pediatric Gastroenterology and Nutrition, Xinhua Hospital, School of Medicine, Shanghai Jiao Tong University, Shanghai, China; 2 Shanghai Key Laboratory of Pediatric Gastroenterology and Nutrition, Shanghai, China; 3 Shanghai Institute of Pediatric Research, Shanghai, China

**Keywords:** B3GNT8, epithelial glycosylation, goblet cells, intestinal barrier, Paneth cells

## Abstract

Emerging evidence suggests that alterations in intestinal epithelial glycosylation are implicated in the pathogenesis of inflammatory bowel disease (IBD). However, the intricate roles of gut glycosylation in maintaining intestinal homeostasis remain inadequately elucidated. Beta 1, 3-N-acetylglucosaminyltransferases (B3GNTs) are Golgi glycosyltransferases involved in the biosynthesis of poly-N-acetyl-lactosamine chains. In this study, we here create *B3gnt8* knockout (*B3gnt8*^*−/−*^) mice to investigate its precise effects on intestinal homeostasis. Our findings reveal that both messenger RNA (mRNA) and protein levels of B3GNT8 are significantly diminished in the inflamed mucosa of pediatric IBD patients. Furthermore, the levels of B3GNT8 were negatively correlated with ulcerative colitis (UC) progression. *B3gnt8*^*−/−*^ mice exhibited heightened vulnerability to Dextran sodium sulfate (DSS)-induced intestinal inflammation, characterized by compromised tight junction integrity and impaired secretion of Mucin from goblet cells. The loss of B3gnt8 resulted in a significant reduction in Paneth cell populations as well as diminished lysozyme content, leading to an altered composition and adhesion properties of intestinal bacteria. Additionally, B3gnt8 deficiency impaired lysosomal stability, potentially reducing glycosylation of lysosomal-associated membrane proteins half (LAMP1/2). From a mechanistic perspective, deficiency in B3gnt8 disrupted autophagy-lysosomal processes within Paneth cells may *via* the ATG16L1-ATG12-ATG5 pathway. Notably, the absence of B3gnt8 rendered these mice more susceptible to DSS-induced colitis. In conclusion, our findings identify B3GNT8 as a key player in intestinal epithelial glycosylation, thereby revealing a potential target for new IBD therapeutics.

The inflammatory bowel disease (IBD), encompassing ulcerative colitis (UC) and Crohn's disease (CD), is a chronic inflammatory disorder of the gastrointestinal tract. They collectively affect an estimated 6.8 million individuals worldwide, with the majority of diagnoses occurring during adolescence and young adulthood (1). In patients with IBD, a convergence of host genetic factors, environmental influences, and microbial interactions leads to inappropriate immune activation in response to gut microbiota (2, 3, 4). A key question remains regarding how genes, environmental factors, and gut microbiota interact to contribute to the pathogenesis of IBD. Emerging evidence suggests that intestinal epithelial glycosylation facilitates interactions between gut microbiota and intestinal epithelia, regulated by both host genetics and environmental conditions (5, 6, 7). Epithelial glycans serve as ligands and nutrients while inducing host signaling pathways that regulate gut microbiota; these processes are altered in IBD (8, 9, 10, 11).

N-linked and O-linked glycans are key components of the cell surface glycocalyx and secreted glycoproteome (12, 13). These glycans are characterized by their specific glycan–protein linkages. Polylactosamine (poly-N-acetyl-lactosamine) is a critical feature found in glycolipids as well as N-linked and O-linked glycans on glycoproteins. This structure consists of multiple repeats of the N-acetyl-lactosamine (Galβ1-4GlcNAc) unit connected through β1-3 linkages, playing a significant role in various biological processes, including immune responses (14, 15). The length of the polylactosamine chain also influences immune reactions; notably, long-chain polylactosamines present on N-glycans have been suggested to suppress excessive immune responses (16, 17, 18). The elongation process for bi-, tri-, and tetra-antennary N-glycans (as well as O-glycans) involves two families of glycosyltransferases: β-1,4-galactosyltransferase (B4GALT) and β-1,3-N-acetylglucosaminyltransferase (B3GNT) (19, 20). These enzymes operate in an alternating manner to incorporate galactose (Gal) and N-acetylglucosamine (GlcNAc) building blocks into the growing chain *via* β1-4 and β1-3 linkages. To date, seven B3GNTs (B3GNT2–B3GNT8), which belong to the family of β1, 3-N-acetylglucosaminyltransferases, have been identified. B3GNT2 utilizes uridine diphosphate (UDP)-activated GlcNAc as a donor sugar to transfer GlcNAc to Gal (the acceptor sugar) in a divalent metal-dependent manner. B3GNT8 has emerged as a novel member within the B3GNT gene family with substrate specificities similar to those of B3GNT2 (21). However, it remains unclear whether B3GNT8 plays a role in the pathogenesis of IBD.

In the present study, we aimed to elucidate the roles and mechanisms of B3GNT8 in maintaining intestinal homeostasis. We commenced by demonstrating that both messenger RNA (mRNA) and protein levels of B3GNT8 are significantly reduced in the inflamed mucosa of pediatric patients with IBD. Following this, we generated *B3gnt8* knockout (*B3gnt8*^*−/−*^) mice to rigorously investigate the specific effects of this gene on intestinal inflammation and barrier integrity, as well as to determine whether alterations in epithelial glycosylation contribute to these mechanisms.

## Results

### B3GNT8 is enriched in gastroenterological tract

As illustrated in Figure S1, B3GNT8 messenger RNA (mRNA) demonstrated a significant enrichment within the gastrointestinal tract, including the small intestines and colon (Fig. S1*A*; Human Protein Atlas proteinatlas.org). Immunohistochemistry (IHC) staining for B3GNT8 revealed its protein expression localized within both the small intestinal and colonic mucosa, exhibiting strong positivity in the cytoplasmic/membranous regions of glandular cells and epithelial cells (Fig. S1*B*; Human Protein Atlas proteinatlas.org). Notably, we confirmed that B3GNT8 protein was predominantly expressed in the mucosa of both pediatric ileum and colon (Fig. S2*A*). Interestingly, B3GNT8 protein was also detected in the Myenteric plexus (Fig. S2*A*). In mice, *B3gnt8* mRNA levels begin to decline in the intestines of mice from embryonic day 12.5 (E12.5), subsequently maintaining relatively stable levels into adulthood (postnatal day zero; P0; Fig. S2*B*). Similarly, the expression pattern of the cell growth marker *Ccnd1* mirrored this temporal trend as indicated (Fig. S2*B*). Within the gastrointestinal tract, single-cell mRNA sequencing revealed that B3GNT8 is specifically expressed in proximal enterocytes, distal enterocytes, Paneth cells, and intestinal goblet cells (Fig. S3; Human Protein Atlas proteinatlas.org).

### Intestinal B3GNT8 is diminished in children with inflammatory bowel disease (IBD)

**Figure 1:**
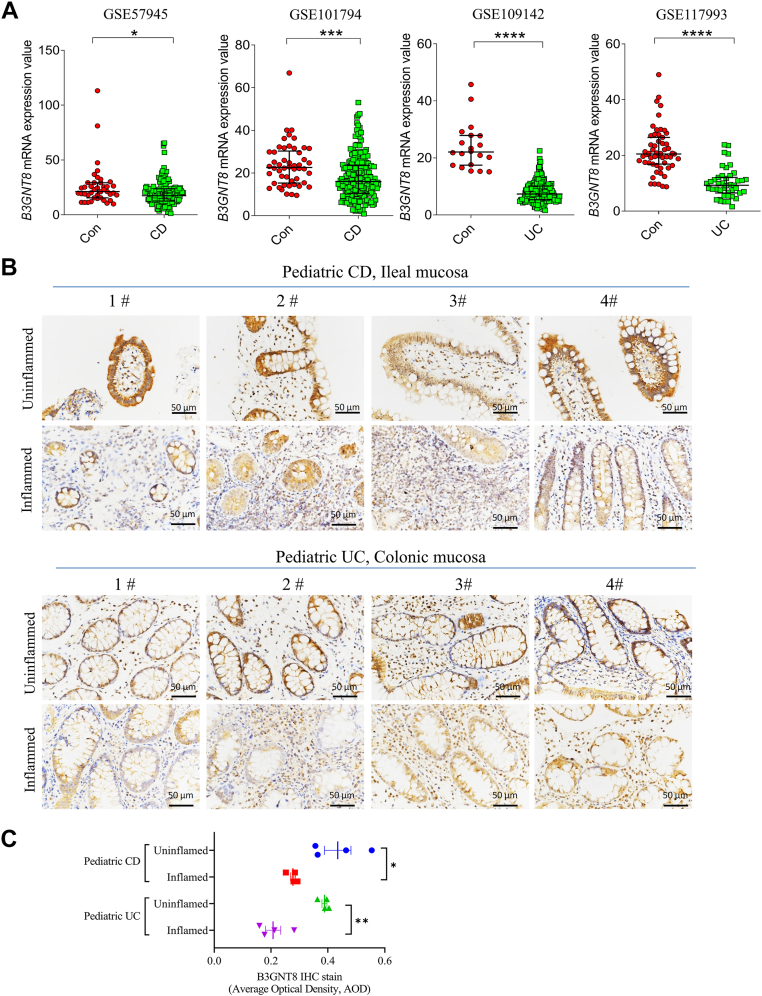
***B3GNT8* expression levels are reduced in diseased intestinal tissues of pediatric Crohn's disease (CD) and ulcerative colitis (UC).***A*, data for *B3GNT8* mRNA expression extracted from the GEO database, comparing ileal tissues from patients with Crohn's disease (CD) or colonic tissues from ulcerative colitis (UC) and non-IBD control subjects. GSE57945, CD, n = 143, control, n = 42; GSE101794, CD, n = 198, control, n = 50; GSE109142, UC, n = 206, control, n = 20, GSE117993, UC, n = 55, control, n = 43. *B*, representative immunohistochemistry (IHC) images of B3GNT8 protein in intestinal tissues of pediatric patients with CD and UC. *C*, quantification of B3GNT8 IHC staining (each group, n = 5). Abbreviation: Con, control; Statistical significance: Unpaired two-tailed Student’s *t* test with Welch’s correction analysis for (*A*, *C*). ∗*p* < 0.05; ∗∗*p* < 0.01; ∗∗∗*p* < 0.001; ∗∗∗∗*p* < 0.0001; ns, not significant.

**Figure 2:**
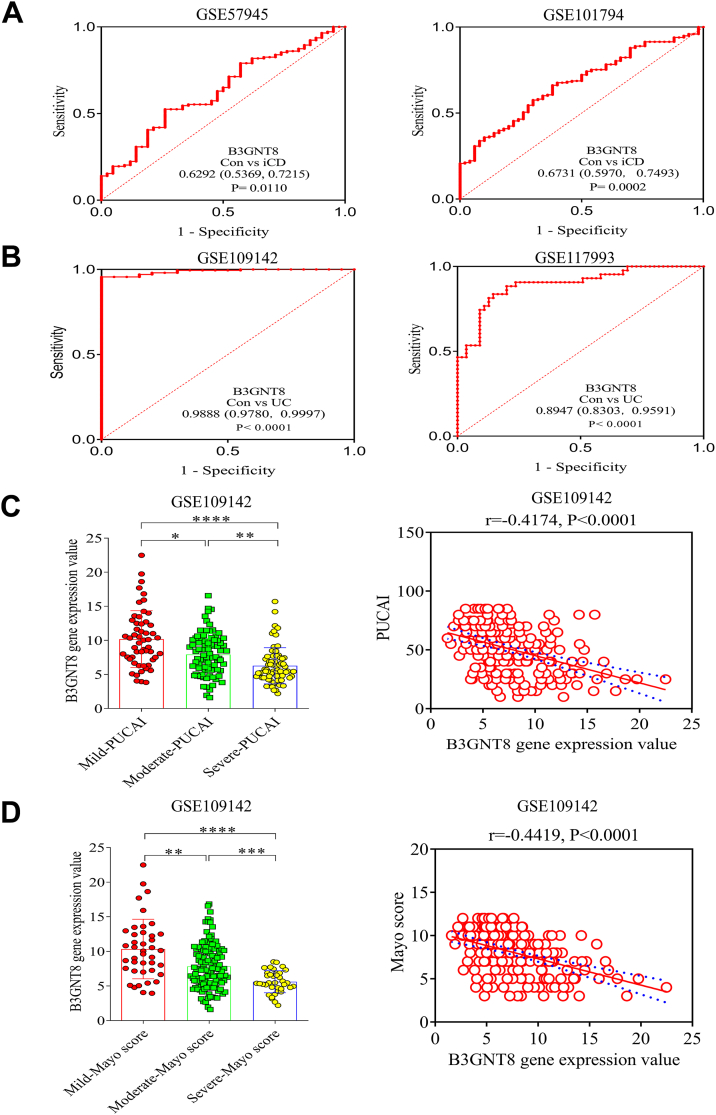
***B3GNT8* expression is correlated with disease severity of pediatric ulcerative colitis (UC).***A* and *B*, the AUC for *B3GNT8* in differentiating UC or CD patients and non-IBD patients. The AUC value is shown with 95% CI. Data were extracted from the datasets GSE57945, CD, n = 143, control, n = 42; GSE101794, CD, n = 198, control, n = 50; GSE109142, UC, n = 206, control, n = 20, GSE117993, UC, n = 55, control, n = 43. *C* and *D*, the mRNA expression levels of *B3GNT8* in UC children with different disease severity. *C*, the mRNA expression levels of *B3GNT8* was correlated with PUCAI in UC children. Data were extracted from the dataset GSE109142. Mild-PUCAI, n = 54; Moderate-PUCAI, n = 83; Severe-PUCAI, n = 69. *D*, the mRNA expression levels of *B3GNT8* was correlated with Mayo scores in UC children. Data were extracted from the dataset GSE109142. Mild- Mayo score, n = 41; Moderate-Mayo score, n = 125; Severe- Mayo score, n = 37; Abbreviation: AUC, area under the curve; CD, Crohn’s disease; CI, confidence interval; PUCAI, pediatric ulcerative colitis activity index; UC, ulcerative colitis. Statistical significance: ANOVA Kruskal-Wallis test and Nonparametric Spearman correlation analysis for (*C*, *D*). ∗*p* < 0.05; ∗∗*p* < 0.01; ∗∗∗*p* < 0.001; ∗∗∗∗*p* < 0.0001.

**Figure 3:**
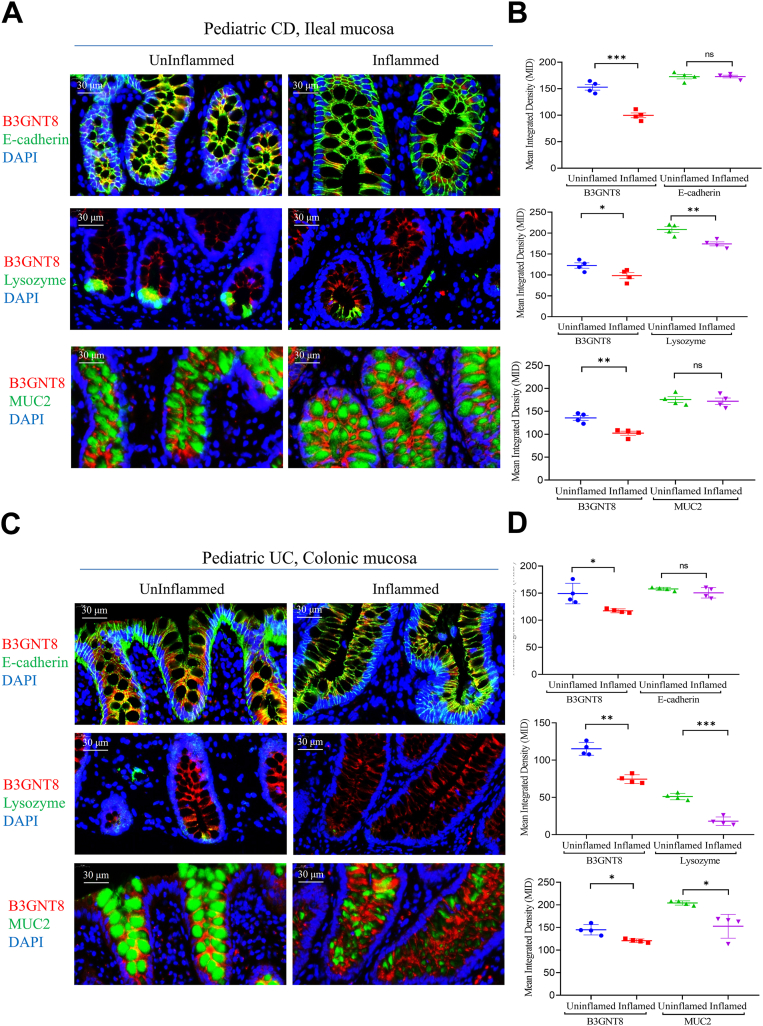
**B3GNT8 protein is reduced in both Paneth cells and goblets cells of pediatric inflammatory bowel disease (IBD).***A*, representative images of immunofluorescence (IF) co-staining between B3GNT8 and E-cadherin, B3GNT8 and Lysozyme, and B3GNT8 and Mucin 2 (MUC2) in ileal mucosa from pediatric patients with Crohn's disease (CD, n = 4). *B*, quantification of *panel* (*A*). *C*, representative images of immunofluorescence (IF) co-staining between B3GNT8 and E-cadherin, B3GNT8 and Lysozyme, and B3GNT8 and Mucin 2 (MUC2) in colonic mucosa from pediatric patients with ulcerative colitis (UC, n = 4). *D*, quantification of *panel* (*C*). Statistical significance: Unpaired two-tailed Student’s *t* test with or without Welch’s correction analysis for (*B*, *D*). ∗*p* < 0.05; ∗∗*p* < 0.01; ∗∗∗*p* < 0.001; ns, not significant. Representative images from n = 4 independent individuals per group are shown. Each data point in the corresponding graphs represents one individual.

Analysis of publicly available datasets revealed that *B3gnt8* mRNA expression levels were significantly reduced in ileal tissues from patients with Crohn's disease (CD) compared to those from control subjects (Fig. 1*A*) (22, 23, 24). Furthermore, the analysis demonstrated a notable decrease in the mRNA expression levels of B3GNT8 in diseased intestinal tissues of children with ulcerative colitis (UC) when compared to control children (Fig. 1*A*) (25, 26). Subsequently, we evaluated the expression of B3GNT8 protein in the intestinal tissues of patients with CD and UC using IHC. As anticipated, our findings indicated a reduction in B3GNT8 protein expression within the inflamed ileal mucosa of patients with CD relative to uninflamed controls (Fig. 1, *B* and *C*). Additionally, lower levels of B3GNT8 protein were observed in the inflamed colonic mucosa of patients with UC compared to their uninflamed counterparts (Fig. 1, *B* and *C*). The area under the curve (AUC) for *B3GNT8* between CD and control groups ranged from 0.5369 to 0.7493 (Fig. 2*A*) (22, 23, 24). In contrast, results from AUCs indicated that B3GNT8 exhibited superior diagnostic value for distinguishing UC from control groups, with AUCs ranging from 0.8303 to 0.9997 (Fig. 2*B*) (25, 26). Moreover, our findings suggested that B3GNT8 mRNA expression levels decreased in rectal tissues as UC severity increased and showed negative correlations with both Pediatric UC Activity Index (PUCAI) scores and total Mayo scores (Fig. 2, *C* and *D*) (25). Immunofluorescence (IF) staining demonstrated a reduced co-expression between B3GNT8 protein and epithelial cells marked by E-cadherin, Paneth cells identified by lysozyme staining, as well as goblet cells characterized by MUC2 expression in inflamed intestinal samples obtained from pediatric cases of CD and UC, compared to their uninflamed counterparts (Fig. 3, *A*–*D*). To delineate the relationship between B3GNT8 and its homolog B3GNT2 in the intestinal tract, we performed immunofluorescence staining. B3GNT2 was detected throughout the intestinal tissues with a broad expression pattern. Notably, B3GNT8 and B3GNT2 exhibited significant co-localization within the mucosal layer, particularly within Paneth cells and goblet cells (Fig. S4).

### B3gnt8 deficiency impaired intestinal homeostasis in mice

To investigate whether *B3gnt8* directly influences intestinal homeostasis, we initially generated mice deficient in the *B3gnt8* gene (*B3gnt8*^*−/−*^, Fig. S5). To confirm the successful CRISPR/Cas9-mediated gene knockout, we genotyped the mice using PCR, which clearly revealed the intended 348 bp deletion at the target locus(Fig. S5*B*). Subsequently, we validated the knockout at the protein level by Western Blot analysis of protein lysates from mouse small intestines. The results demonstrated a complete absence of the B3GNT8 protein band in homozygous knockout mice compared to their *Wild-type (Wt)*, functionally confirming the successful establishment of the model (Fig. S5*C*). The *B3gnt8*^*−/−*^ mice were readily observable for their overall growth retardation and unkempt fur compared with their *Wt* littermates of the same age (Fig. S5*D*). Histopathological examination revealed widespread tissue damage of varying severity in the knockout mice, notably featuring inflammatory cell infiltration in vital organs such as the liver, lung, brain, kidney, and pancreas (Fig. S5*E*).

**Figure 4:**
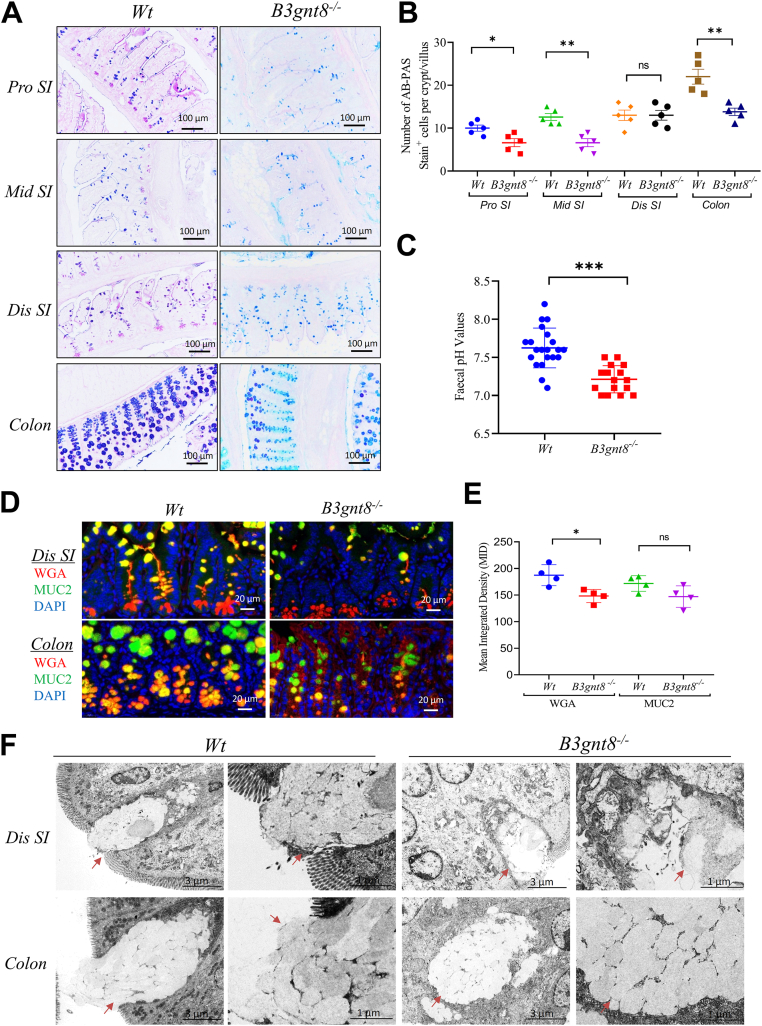
***B3gnt8* knockout (*B3gnt8*^*−/−*^) impaired goblet cells functions.***A*, representative images of Alcian blue/periodic acid Schiff base (AB-PAS) staining for the proximal (pro), middle (mid), and distal (dis) small intestines (SI) from both *B3gnt8*^*−/−*^ mice and *Wild*-*type* (*Wt*) mice. *B*, quantification of goblet cells number (stained *blue*) in both *B3gnt8*^*−/−*^ mice (n = 5) and *Wt* mice (n = 5). *C*, measuring the fecal pH values of *B3gnt8*^*−/−*^ mice (n = 16) and *Wt* mice (n = 21). *D*, representative images of immunofluorescence (IF) co-staining between Wheat germ agglutinin (WGA) and Mucin 2 (MUC2) in distal (dis) small intestines (SI) and colons from both *B3gnt8*^*−/−*^ mice (n = 4) and *Wild*-*type* (*Wt*) mice (n = 4). *E*, quantification of positive cells in *panel* (*D*). *F*, representative images of Transmission electron microscopy (TEM) for goblet cells in both small intestines (SI) and colons from both *B3gnt8*^*−/−*^ mice and *Wt* mice. *Arrows* indicated goblet cells. Statistical significance: Unpaired two-tailed Student’s *t* test with or without Welch’s correction analysis for (*B*, *C*, *E*). ∗*p* < 0.05; ∗∗*p* < 0.01; ∗∗∗*p* < 0.001; ns, not significant.

In addition, the knockout mice exhibited intestinal abnormalities, notably more fragile intestinal tissue and a statistically significant shortening of the colon compared to the control group (Fig. S6, *A* and *B*). Histological analysis revealed that *B3gnt8*^*−/−*^ mice exhibited higher pathological scores in their intestines compared to *Wt* controls (Fig. S6, *C* and *D*). Furthermore, Alcian Blue-Periodic Acid-Schiff (AB-PAS) staining demonstrated a significant reduction in goblet cells within both the small intestines and colons of *B3gnt8*^*−/−*^ mice when compared to those from *Wt* mice, and the goblet cells appeared more intensely stained blue (acid) in *Wt* mice (Fig. 4, *A* and *B*). Notably, fecal pH values were significantly lower in *B3gnt8*^*−/−*^ mice relative to their *Wt* counterparts (Fig. 4*C*). Co-staining with MUC2 (green) and wheat germ agglutinin (WGA, red), which selectively binds to N-acetylglucosaminyl (GlcNAc) and N-acetylneuraminic acid residues on glycoconjugates and oligosaccharides, indicated a decrease of WGA binding in MUC2-positive goblet cells of *B3gnt8*^*−/−*^ mice (Fig. 4, *D* and *E*). Transmission electron microscopy (TEM) further illustrated damaged goblet cells along with abnormal mucus secretion from these cells in *B3gnt8*^*−/−*^ mice (Fig. 4*F*).

**Figure 5:**
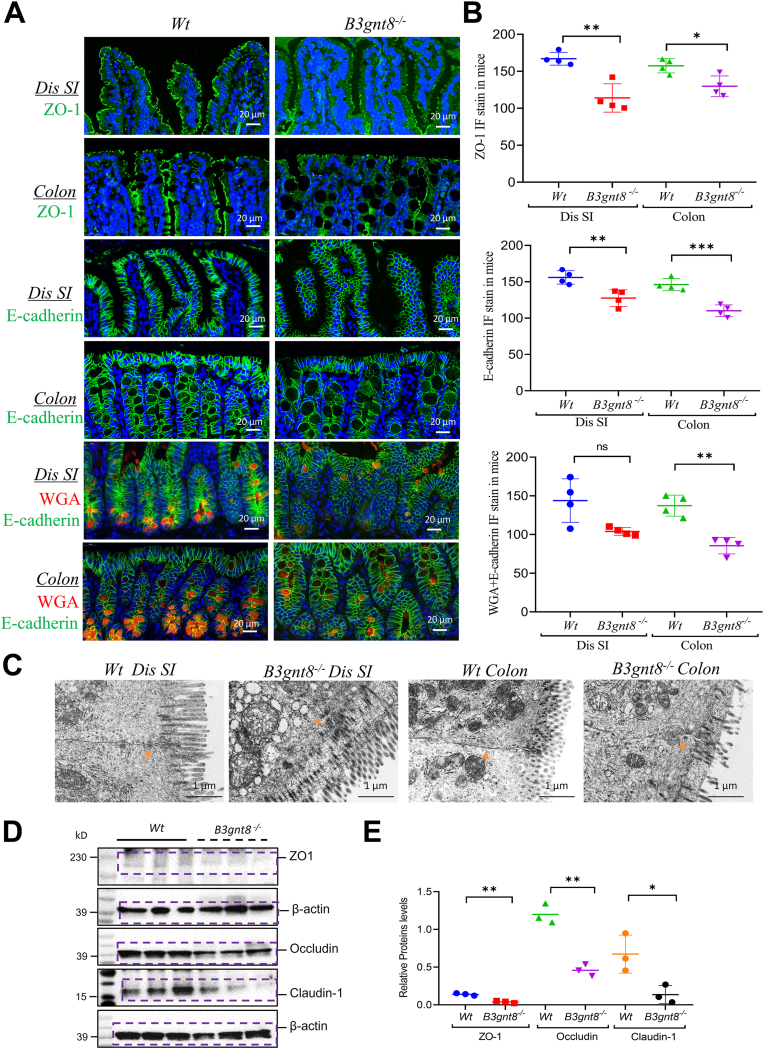
***B3gnt8* knockout (*B3gnt8*^*−/−*^) injured intestinal tight junctions.***A*, representative images of immunofluorescence (IF) staining of Zonula occludens-1 (ZO-1, (*green*), E-cadherin (*green*) and wheat germ agglutinin (WGA, *red*) for the distal (dis) small intestines (SI) and colons from both *B3gnt8*^*−/−*^ mice (n = 4) and *Wild*-*type* (*Wt*) mice (n = 4). *B*, quantification of ZO-1, E-cadherin and WGA in *panel* (*A*). *C*, transmission electron microscopy (TEM) analysis tight junctions in intestines of *B3gnt8*^*−/−*^ mice and *Wt* mice. *D*, representative images of western blotting (WB) analysis for tight junctions proteins, ZO-1, Occludin, and Claudin-1 in distal (dis) small intestines (SI) mucosa of *B3gnt8*^*−/−*^ mice and *Wt* mice (each group, n = 3). *E*, quantification of ZO-1, E-cadherin and WGA proteins levels in *panel* (*D*). Statistical significance: Unpaired two-tailed Student’s *t* test with or without Welch’s correction analysis for (*B*, *E*). ns, not significant; ∗*p* < 0.05; ∗∗*p* < 0.01. Representative images from n = 4 independent individuals per group are shown. Each data point in the corresponding graphs represents one individual.

The immunofluorescence (IF) staining revealed a significant decrease in the expression of tight junction protein zonula occludens-1 (ZO-1) in both the small intestines (SI) and colons of *B3gnt8*^*−/−*^ mice compared to their *Wt* counterparts (Fig. 5, *A* and *B*). Additionally, IF staining indicated that the levels of adherent junction protein epithelial cadherin (E-cadherin) were also reduced in both the small intestines and colons of *B3gnt8*^*−/−*^ mice relative to those observed in *Wt* mice (Fig. 5, *A* and *B*). Furthermore, wheat germ agglutinin (WGA) staining was diminished in the epithelial cells of *B3gnt8*^*−/−*^ mice when compared to *Wt* controls (Fig. 5, *A* and *B*). Transmission electron microscopy (TEM) analysis revealed damage to intercellular junctions among intestinal epithelial cells, along with irregular distribution of microvilli in *B3gnt8*^*−/−*^ mice as opposed to their *Wt* littermates (Fig. 5*C*). Moreover, Western blotting (WB) analysis demonstrated a marked reduction in the expression levels of tight junction proteins ZO-1, Occludin, and Claudin-1 within the small intestines from *B3gnt8*^*−/−*^ mice compared to those derived from their *Wt* counterparts (Fig. 5, *D* and *E*).

### B3gnt8 knockout reduces Paneth cells with impairing autophagy

**Figure 6:**
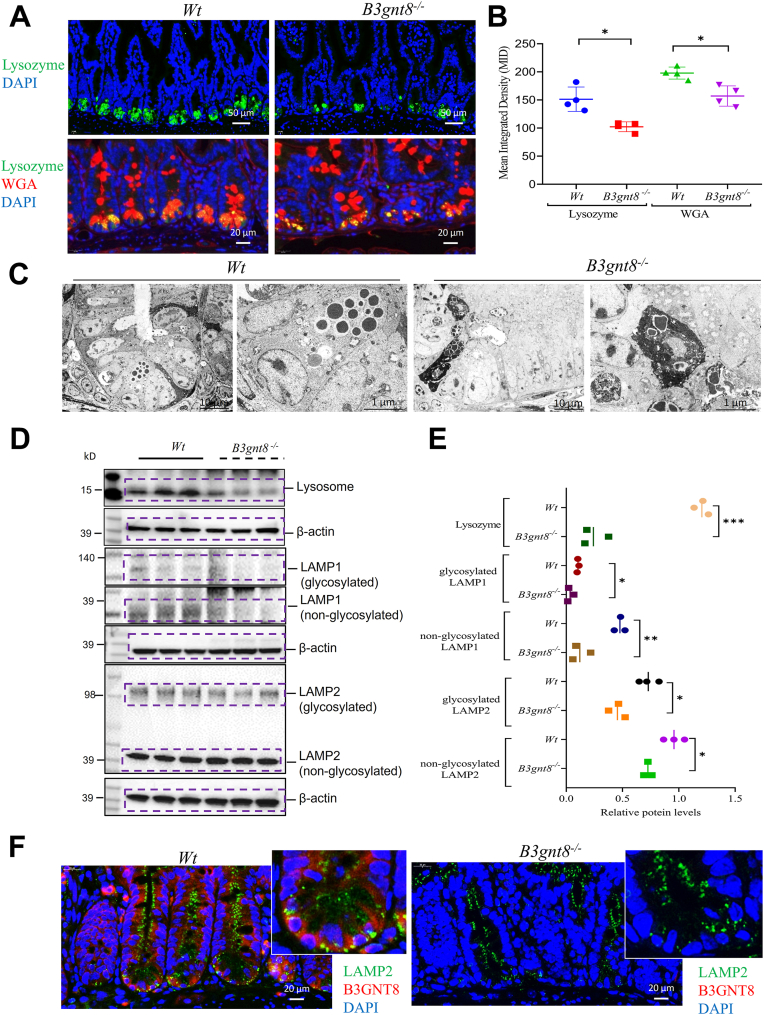
***B3gnt8* deficiency impaired Paneth cells.***A*, representative images of immunofluorescence (IF) staining of Lysozyme (*green*) and wheat germ agglutinin (WGA, *red*) for the distal (dis) small intestines (SI) from both *B3gnt8* knockout (*B3gnt8*^*−/−*^) mice (n = 4) and *Wild*-*type* (*Wt*) mice (n = 4). *B*, quantification of Lysozyme and WGA in *panel* (*A*). *C*, representative images of Transmission electron microscopy (TEM) for Paneth cells in distal (dis) small intestines (SI) from both *B3gnt8*^*−/−*^ mice and *Wt* mice. *D*, representative images of western blotting (WB) analysis for Lysozyme markers proteins, Lysozyme, LAMP1 and LAMP2 in distal (dis) small intestinal mucosa of *B3gnt8*^*−/−*^ mice and *Wt* mice (each group, n = 3). *E*, quantification of Lysozyme, LAMP1 and LAMP2 proteins levels in *panel* (*D*). *F*, representative images of immunofluorescence (IF) staining of LAMP2 (*green*) and B3GNT8 (*red*) for the distal (dis) small intestines (SI) from both *B3gnt8* knockout (*B3gnt8*^*−/−*^) mice and *Wild*-*type* (*Wt*) mice. Statistical significance: Unpaired two-tailed Student’s *t* test with or without Welch’s correction analysis for (*B*, *E*). ∗*p* < 0.05; ∗∗*p* < 0.01; ∗∗∗*p* < 0.001.

Immunofluorescence (IF) staining-based detection of lysozyme-expressing cells confirmed that the count of Paneth cells and WGA staining were both significantly reduced in the small intestinal mucosa of *B3gnt8*^*−/−*^ mice compared to their *Wt* littermates (Fig. 6, *A* and *B*). Additionally, representative transmission electron microscopy (TEM) further illustrated that *B3gnt8*^*−/−*^ mice exhibited fewer and smaller granules (Fig. 6*C*). Western blotting (WB) analysis demonstrated a decline in key markers associated with Paneth cells, including lysozyme as well as lysosomal markers LAMP1 and LAMP2, particularly glycosylated forms of LAMP1 and LAMP2, within the intestinal mucosa of *B3gnt8*^*−/−*^ mice (Fig. 6, *D* and *E*). Moreover, representative IF staining revealed that B3GNT8 co-localized with LAMP2. Notably, levels of LAMP2 were diminished in the absence of *B3gnt8* (Fig. 6*F*).

**Figure 7:**
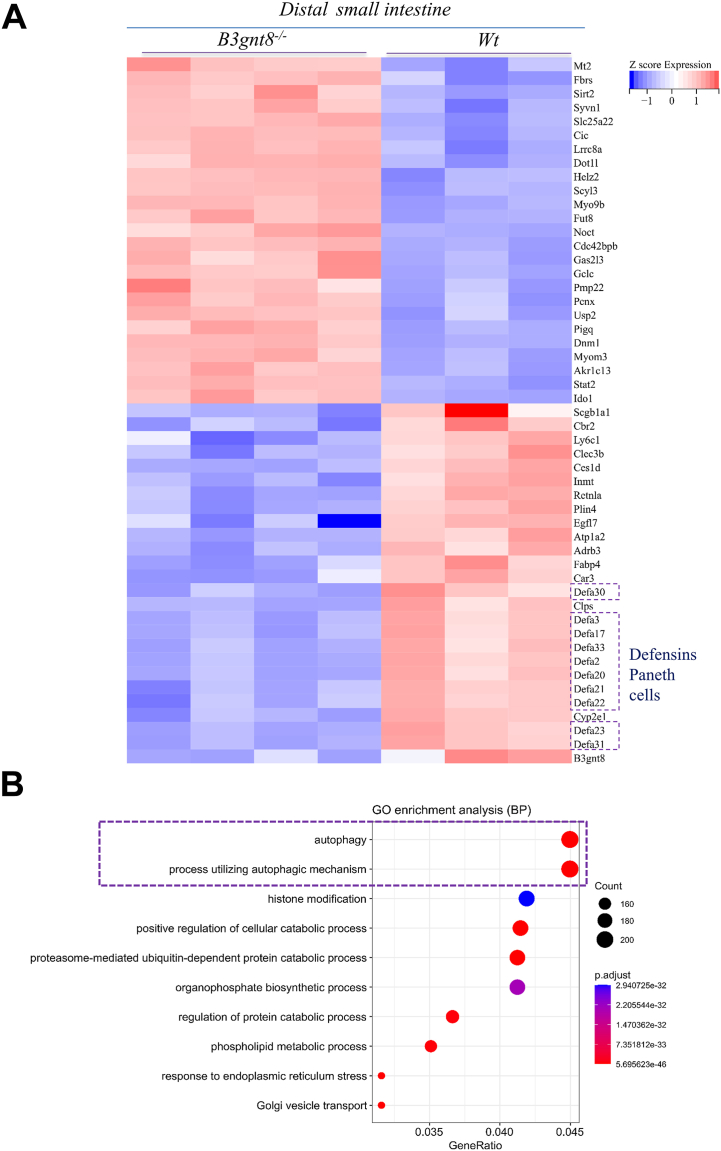
**The differentially expressed genes in the distal small intestinal mucosa of *B3gnt8* knockout (*B3gnt8*^*−/−*^) mice and *Wild***-***type* (*Wt*) mice.***A*, heatmap of differentially expressed genes in the distal small intestines of *B3gnt8*^*−/−*^ mice (n = 4) and *Wt* mice (n = 3). *B*, Gene Ontology (GO) enrichment analysis results of differentially expressed genes in the small intestines of *B3gnt8*^*−/−*^ mice and *Wt* mice.

**Figure 8:**
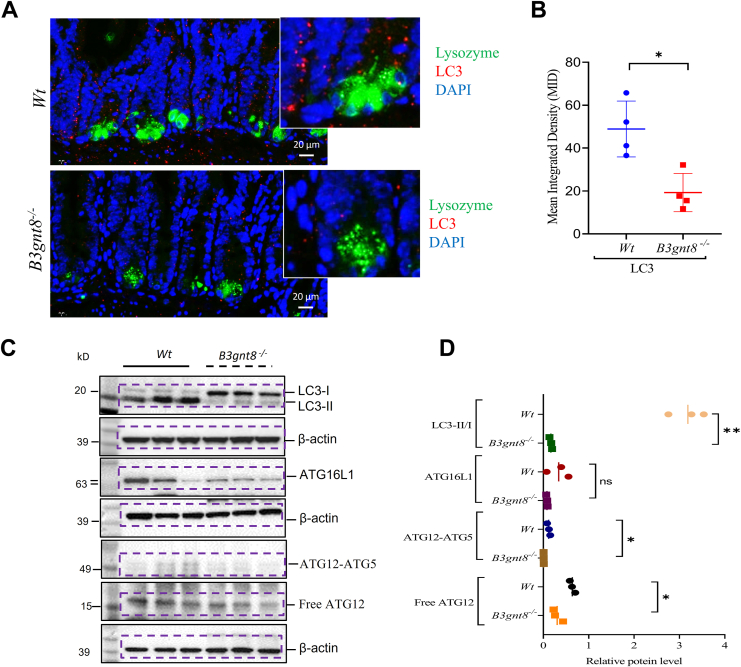
***B3gnt8* deficiency impaired the autophagy in Paneth cells.***A*, representative images of Immunofluorescence analysis for LC3 and Lysosome in the sections of distal (dis) small intestine of knockout (*B3gnt8*^*−/−*^) mice and *Wild*-*type* (*Wt*) mice. *B*, quantification of LC3 and Lysozyme positive cells in *panel (A)* (each group, n = 4). *C*, the Western blotting (WB) analysis was used to determine the expression levels of LC3, ATG16L1, ATG12-ATG5 in the distal (dis) small intestinal mucosa of *B3gnt8*^*−/−*^ mice and *Wt* mice. Each group, n = 3. *D*, quantification of them against β-actin in panel (*C*). Independent experiments at least two times. Statistical significance: Unpaired two-tailed Student’s *t* test with or without Welch’s correction analysis for (*B*, *D*); ns, not significant; ∗*p* < 0.05; ∗∗*p* < 0.01.

To investigate the pathways influenced by *B3gnt8* in Paneth cells, we initially employed RNA sequencing to elucidate genes affected within the distal small intestinal mucosa. In distal small intestines of *B3gnt8*^*−/−*^ mice, our findings indicated a downregulation of genes associated with defensins secreted by Paneth cells (Fig. 7*A*). Comprehensive analysis suggested that within this region of the distal small intestinal mucosa, the most significantly altered genes were notably enriched in processes related to autophagy and mechanisms utilizing autophagic pathways (Fig. 7*B*). Indeed, IF staining demonstrated that *B3gnt8*^*−/−*^ mice had lower puncta levels for MAP1LC3 (LC3), an established marker for autophagy, in their distal small intestinal mucosa compared to *Wt* controls (Fig. 8, *A* and *B*). Furthermore, colocalization puncta between LC3 and lysozyme were found to be reduced in *B3gnt8*^*−/−*^ mice relative to those observed in *Wt* counterparts (Fig. 8, *A* and *B*). WB confirmed that the expression of LC3-II was significantly reduced in the distal small intestinal mucosa of *B3gnt8*^*−/−*^ mice compared to that of *Wt* mice (Fig. 8, *C* and *D*). Atg16L1 has been identified as a functional link between two crucial ubiquitin-like conjugations systems involved in autophagy, wherein Atg16L1 binds to Atg5 within the Atg12-Atg5 conjugate, thereby forming a complex (27). Atg12-Atg5-Atg16L1 complex localizes to pre-autophagosomal membranes, determining the site for LC3-II lipidation and catalyzing the reactions necessary for mature autophagosome formation. (28). WB demonstrated that the Atg12-Atg5-Atg16L1 pathway was downregulated in the distal small intestinal mucosa of *B3gnt8*^*−/−*^ mice compared to those of *Wt* mice (Fig. 8, *C* and *D*).

### B3gnt8 deficiency alters intestinal bacteria defensing in mice

**Figure 9:**
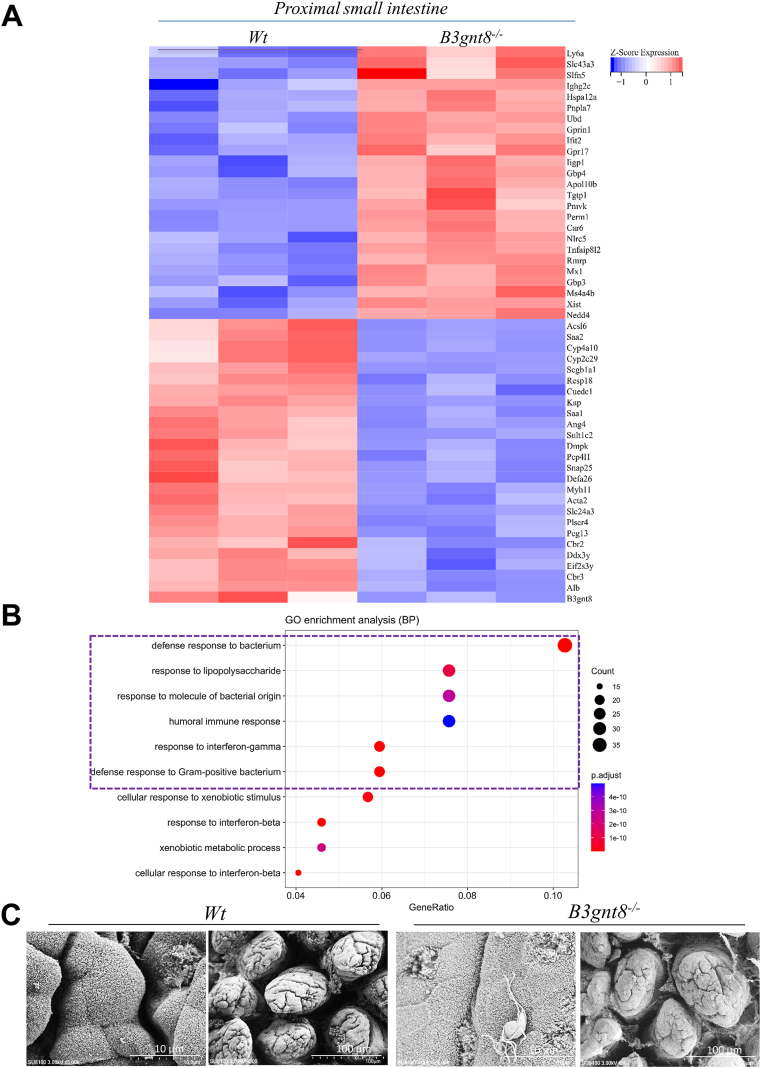
**The differentially expressed genes in the proximal (pro) small intestinal mucosa of *B3gnt8* knockout (*B3gnt8*^*−/−*^) mice and *Wild***-***type* (*Wt*) mice.***A*, Heatmap of differentially expressed genes in the proximal (pro) small intestines of *B3gnt8*^*−/−*^ mice (n = 3) and *Wt* mice (n = 3). *B*, gene Ontology (GO) enrichment analysis results of differentially expressed genes in the proximal (pro) small intestines of *B3gnt8*^*−/−*^ mice and *Wt* mice. *C*, representative images of scanning electron microscopy (SEM) analysis for the proximal (pro) small intestinal mucosa of both *B3gnt8*^*−/−*^ and *Wt* mice.

**Figure 10:**
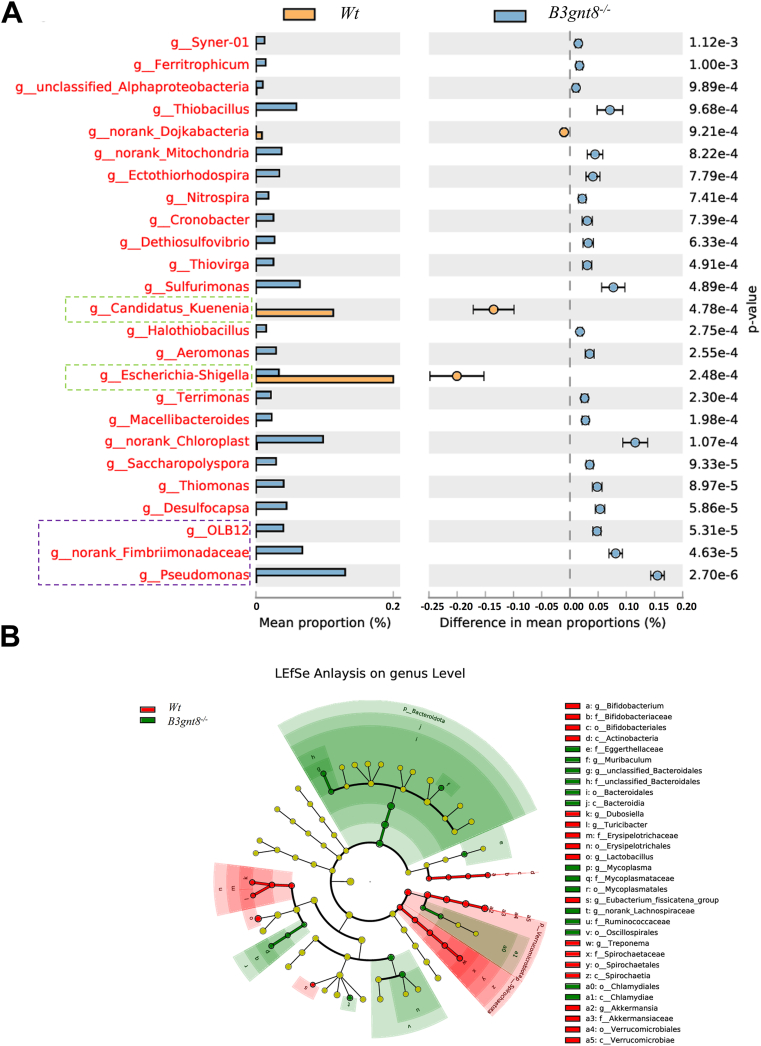
***B3gnt8* deficiency altered the bacteria composition.***A*, the relative abundance of the top bacteria (genus) in the feces of *B3gnt8* knockout (*B3gnt8*^*−/−*^) mice (n = 5) and *Wild*-*type* (*Wt*) mice (n = 5). *B*, lineage-specific effect size (LEfSe) analysis for the most discriminative bacterial clades between *B3gnt8*^*−/−*^ mice (n = 5) and *Wt* mice (n = 5).

RNA sequencing analysis revealed that the most significantly altered genes were notably enriched in processes associated with defense responses against bacteria, as well as responses to lipopolysaccharide and bacterial origins. This was observed in the proximal (pro) small intestines of *B3gnt8*^*−/−*^ mice compared to their *Wt* littermates (Fig. 9, *A* and *B*). Furthermore, scanning electron microscopy (SEM) analysis demonstrated a marked increase in the number of invading bacteria adhering to and aggregating on the epithelial surface within the proximal small intestines of *B3gnt8*^*−/−*^ mice (Fig. 9*C*). Subsequently, we employed 16S rRNA sequencing analysis to investigate intestinal bacterial composition in both *B3gnt8*^*−/−*^ and *Wt* mice (Fig. S7). In comparison with their *Wt* littermates, *B3gnt8*^*−/−*^ mice exhibited a higher abundance of operational taxonomic units (OTUs) in the genera *Pseudomonas*, *Fimbriimonadaceae*, *OLB12*, among others in feces, conversely, there was a reduction in *Escherichia-Shigella* and *Candidatus-Kuenenia* abundances within these samples (Fig. 10*A*). A cladogram illustrating the most discriminative bacterial clades between *B3gnt8*^*−/−*^ and *Wt* mice identified through LEfSe analysis is presented. Regions highlighted in green indicate clades that like Eggerthellaceae, *Bacteroidales* were enriched in *B3gnt8*^*−/−*^ mice, while regions marked in red denote clades that were more abundant in *Wt* mice (Fig. 10*B*). Moreover, IHC staining revealed an increase in CD68^+^ macrophages along with elevated levels of lymphocyte-specific transcription factors TBX21 (Th1) within the small intestines of *B3gnt8*^*−/−*^ mice; however, Ki-67-positive proliferative cells were found to be reduced within the small intestines of these same animals (Fig. S8).

### Loss of B3gnt8 aggravates dextran sulfate sodium (DSS)-induced colitis in mice

**Figure 11:**
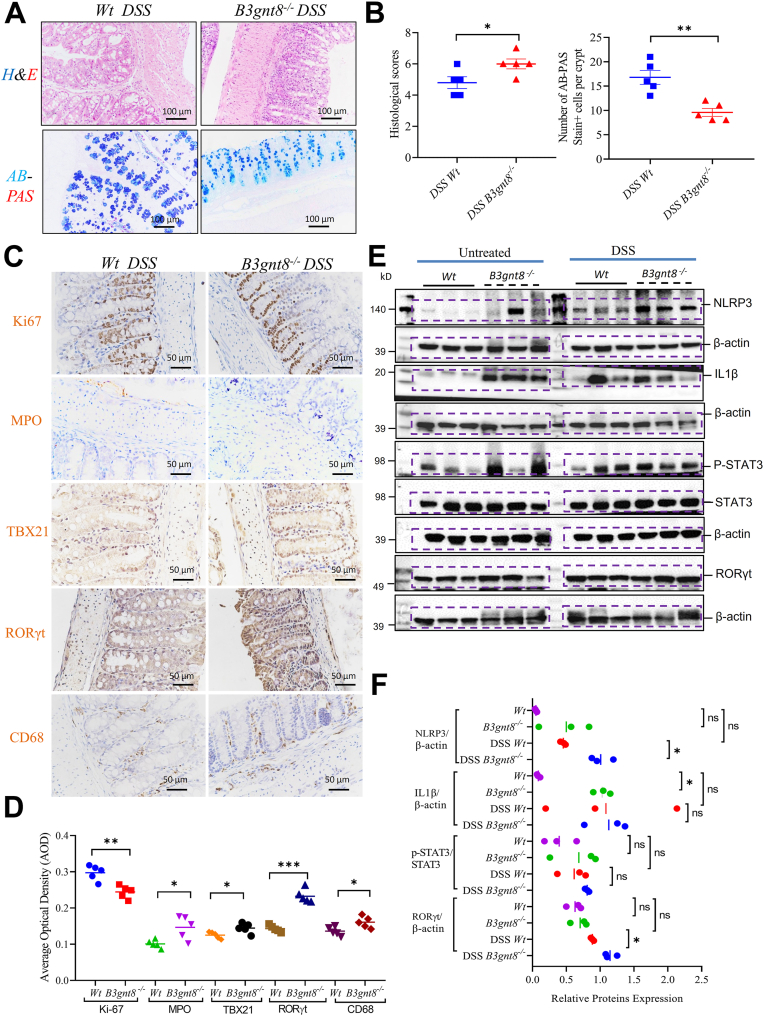
***B3gnt8 deficiency* worsen dextran sulfate sodium (DSS)-induced colitis in mice.***A*, representative images of Hematoxylin and Eosin (H&E) and Alcian blue/periodic acid Schiff base (AB-PAS) staining colons from dextran sulfate sodium (DSS)-treated *B3gnt8* knockout (*B3gnt8*^*−/−*^) mice (n = 5) and DSS *Wild*-*type* (*Wt*) mice (n = 5). *B*, quantification of histological scores and AB-PAS staining in *panel (A*). *C*, representative images of Ki-67, Myeloperoxidase (MPO), TBX21, RORγt and CD68 immunochemistry stain (IHC) in colons from both DSS *B3gnt8*^*−/−*^ (n = 5) mice and DSS *Wt* (n = 5) mice. *D*, quantification of them in *panel (C*). *E*, representative images of western blotting (WB) analysis for NLRP3, IL-1β, phosphorylation STAT3 (p-STAT3), and RORγt proteins in colonic mucosa of *B3gnt8*^*−/−*^ mice and *Wt* mice with or without DSS treatment (each group, n = 3). *F*, quantification of proteins levels in *panel (E*). Statistical significance: Unpaired two-tailed Student’s *t* test with or without Welch’s correction analysis for (*B*), (*D*), and (*F*). ns, not significant, ∗*p* < 0.05, ∗∗*p* < 0.01, ∗∗∗*p* < 0.001.

RNA sequencing analysis revealed that the most significantly altered genes were notably enriched in processes associated with histone modification, cellular catabolic processes, cell cycle regulation, and autophagy in the colons of *B3gnt8*^*−/−*^ mice compared to their *Wt* littermates (Fig. S9). To determine whether these transcriptomic alterations in key homeostatic pathways translate to a functional phenotype, we challenged the mice with dextran sulfate sodium (DSS). Following DSS exposure, the *B3gnt8*^*−/−*^ mice displayed an exacerbated disease phenotype relative to *Wt* counterparts. This was characterized by greater weight loss, increased severity of colonic congestion and edema, and a statistically significant reduction in colon length (Fig. S10, *A*–*C*). In our DSS-induced colitis mouse model, colon histopathological examination indicated that *B3gnt8*^*−/−*^ mice exhibited greater mucosal damage and increased inflammatory infiltration than DSS-treated *Wt* mice (Fig. 11, *A* and *B*). DSS-induced inflammatory damage was not confined to the colon but also occurred in the small intestine, where it was significantly exacerbated in *B3gnt8*^*−/−*^ mice (Fig. S10, *D* and *E*). Importantly, the number of Ki67-positive cells was significantly reduced within the colonic mucosa of *B3gnt8*^*−/−*^ mice (Fig. 11, *C* and *D*). Furthermore, levels of CD68, TBX21, and RORγt were elevated in the colonic mucosa of DSS-treated *B3gnt8*^*−/−*^ mice compared to DSS-treated *Wt* counterparts (Fig. 11, *C* and *D*). Consistent with these histological findings, Western blotting analysis demonstrated an upregulation in the expression levels of inflammatory proteins—including NLRP3, IL-1β, phosphorylated STAT3, and IL-17 regulator RORγt—in the colonic mucosa of *B3gnt8*^*−/−*^ mice following DSS treatment when compared to their DSS-treated *Wt* littermates (Fig. 11, *E* and *F*).

## Discussion

To the best of our knowledge, this study proposes that B3GNT8 acts as a glycosyltransferase essential for intestinal homeostasis. We are the first to show that B3GNT8 protein is enriched in the gastrointestinal tract and expressed in intestinal epithelial cells, including Paneth and goblet cells. In humans, both mRNA and protein levels of B3GNT8 significantly decrease in the intestinal mucosa of pediatric inflammatory bowel disease (IBD) patients. The deficiency of *B3gnt8* disrupts intestinal homeostasis in mice, leading to compromised tight junctions, reduced goblet cell numbers and secretions, and altered gut microbiota composition. Additionally, the absence of *B3gnt8* increases susceptibility to dextran sulfate sodium (DSS)—potentially through impaired autophagy in Paneth cells and disrupted tight junction integrity.

Altered epithelial glycosylation is implicated in the pathogenesis of IBD. Evidence includes disruptions in mucin-type O-glycans, N-glycans, and common termini across various glycosylation classes (7, 29, 30). Glycosylation is a common post-translational modification found in various organisms, playing key biological roles (31). Beta-1,3-N-acetylglucosaminyltransferases (B3GNTs), a subgroup of glycosyltransferases, are linked to the onset and progression of several diseases, including autoimmune disorders, cancers, neurodevelopmental conditions, musculoskeletal disorders, and metabolic diseases (32). The role of B3GNTs highlights that protein glycosylation is a critical and potentially life-threatening factor in many diseases. Among these enzymes, B3GNT8 is notably enriched in the gastrointestinal tract and esophagus; however, its exact functions are largely unknown. Thus, B3GNT8 performs an indispensable, non-redundant function in gut homeostasis that is not fully supported by its homologs. Given this non-redundant function, our study reveals that B3GNT8 expression is significantly reduced in pediatric patients with Crohn's disease (CD) and ulcerative colitis (UC). Our evidence shows that B3GNT8 is mainly expressed in intestinal goblet and Paneth cells. It has been reported that B3GNT2 and B3GNT8 may function synergistically. Our data are in line with this notion, as we observed co-expression of B3GNT2 and B3GNT8 in the intestinal mucosa, particularly within Paneth cells and goblet cells. This spatial co-localization suggests that B3GNT2 might provide limited compensatory action in the absence of B3GNT8. To test this hypothesis, we generated B3gnt8 knockout mice. The pronounced inflammatory and damage phenotypes observed in the *B3gnt8*^*−/−*^ mice unequivocally demonstrate that any compensatory capacity of B3GNT2 is severely limited. Consequently, our findings establish a non-redundant and critical role for B3GNT8 in maintaining intestinal homeostasis (21, 33). Epithelial glycosylation is crucial for barrier formation, host-microorganism symbiosis, and immune responses. Thus, altered glycosylation likely plays a significant role in inflammatory bowel disease (IBD). Mouse models indicate that glycans are essential for synthesizing the MUC2 mucus layer from goblet cells, which forms a loose layer in the small intestine and both an outer loose layer and an inner attached layer in the colon (34). The loose layers in the small intestine and colon provide habitats and nutrients for gut microbiota while allowing bacterial permeability. In contrast, the inner mucus layer is impermeable to bacteria, preventing bacterial–epithelial interactions in the distal gut (34). In this study, we showed that *B3gnt8* knockout reduced goblet cells and altered MUC2 secretion. As a result, the loss of *B3gnt8* increased fecal acidity; lower pH levels are linked to IBD patients with similar traits (35). Furthermore, 16S rRNA sequencing revealed an increase in several operational taxonomic units (OTUs) from genera like *Pseudomonas*, *Fimbriimonadaceae*, and *OLB12* in *B3gnt8*^*−/−*^ mice compared to wild-type littermates. Scanning electron microscopy (SEM) showed that *B3gnt8*^*−/−*^ mice had more invasive bacteria adhering to the intestinal epithelial surface. Intestinal epithelial glycosylation regulates gut microbiota composition; thus, altered glycosylation contributes to dysbiosis patterns seen in IBD (5). Our findings show that *B3gnt8* deficiency impairs intestinal tight junction proteins by reducing ZO-1, Claudin-1, Occludin, and E-cadherin levels. Additionally, intestinal epithelial glycosylation was significantly reduced in *B3gnt8*^*−/−*^ mice. These results suggest that B3GNT8 is crucial for maintaining the integrity of the intestinal barrier through its role in epithelial glycosylation.

Paneth cells, located at the bases of intestinal crypts, play a crucial role in protecting the intestinal environment from enteropathogens by constitutively secreting a diverse array of antimicrobial peptides (AMPs) and bactericidal proteins (36, 37). A comprehensive single-cell sequencing analysis has revealed that B3GNT8 is highly expressed in Paneth cells, suggesting its potential functional significance for these specialized cells. Indeed, our study demonstrated that the loss of B3gnt8 resulted in a reduction in the number of intestinal Paneth cells and impaired lysozyme secretion. Further evidence indicated that B3gnt8 contributes to the stability of lysozyme through glycosylation of lysosomal membrane proteins LAMP1 and LAMP2. Autophagy deficiency within the intestinal milieu has been reported to lead to aberrant morphological changes in Paneth cells (38, 39, 40). In this study, we observed that *B3gnt8* deficiency inhibited autophagic processes in Paneth cells, as evidenced by reduced LC3 expression. ATG16L1 is an essential autophagy protein that forms a complex with ATG12-ATG5 to mediate autophagy processes in Paneth cells (38, 41). Our findings elucidated that loss of B3gnt8 led to a significant reduction in activity within the ATG16L1-ATG12-ATG5 pathway and compromised their autophagic function. In the DSS induced colitis models mimicking ulcerative colitis (UC) in humans, we observed that loss of B3gnt8 exacerbated DSS-induced intestinal inflammation by reducing cell proliferation and promoting colitis within mouse colon tissues.

## Conclusion

Our findings show that B3GNT8 is downregulated in pediatric IBD. The lack of B3gnt8 damages the intestinal barrier, disrupts microbiota balance, and increases bacterial invasion. These changes heighten susceptibility to intestinal inflammation and alter epithelial glycosylation. Additionally, B3gnt8 impairs Paneth cell functions by disrupting the ATG16L1-ATG12-ATG5 autophagy pathway. Overall, these results suggest that B3GNT8 is essential for maintaining Paneth cell function and intestinal homeostasis through its glycosylation activities, potentially leading to new therapeutic strategies for IBD.

## Experimental procedures

### Analysis of B3GNT8 expression in pediatric inflammatory bowel disease (IBD)

In an effort to elucidate the expression of the B3GNT8 gene among children affected by Crohn’s disease (CD) or ulcerative colitis (UC), we initially utilized public datasets from the Gene Expression Omnibus (GEO) database for comprehensive reanalysis. The datasets related to CD were derived from previous studies and are accessible *via* GEO accession numbers GSE57945 (22) and GSE101794 (23). Specifically, dataset GSE57945 includes an extensive gene expression profile obtained from human ileal tissues of patients diagnosed with ileal CD (n = 143) alongside non-IBD controls (n = 42). Similarly, dataset GSE101794 presents gene expression data from ileal tissues of individuals with ileal CD (n = 198) and non-IBD controls (n = 50). For UC studies, datasets are available through GEO accession numbers GSE109142 (25) and GSE117993 (25, 42). Dataset GSE109142 features a detailed gene expression profile of rectal tissues from pediatric UC patients (n = 206) compared to control samples (n = 20). Dataset GSE117993 provides similar data for pediatric UC patients (n = 43) against control samples (n = 55). To evaluate the diagnostic potential of B3GNT8 for both CD and UC in children, we employed receiver–operating characteristic curves (ROCs) along with area under the curve values (AUCs). We also assessed the correlation between B3GNT8 levels and the pediatric ulcerative colitis activity index (PUCAI) as well as Mayo Scores to explore its relationship with disease severity.

In this study, intestinal mucosa samples were collected from our hospital, including five cases each of pediatric CD and UC, along with five corresponding control specimens. The protein expression levels of B3GNT8 in these samples were thoroughly analyzed. Written informed consent was obtained from legal guardians before participation. Our protocol involving human subjects received ethical approval from the. All procedures involving human participants in this study were performed in accordance with the ethical standards of the Faculty of Medicine's Ethics Committee at Xin Hua Hospital (reference number XHEC-D-2022–030) and with the Declaration of Helsinki.

### Generation of B3gnt8 knockout (B3gnt8^−/−^) mice

*B3gnt8* knockout *(B3gnt8*^*−/−*^*)* (Δexons 1–2) mice were generated using genome engineering mediated by clustered regularly interspaced short palindromic repeats (CRISPRs) and CRISPR-associated protein 9 (Cas9) in C57BL/6J mice. *B3gnt8* transcript (ENSMUST00000076034.7) is served as the knockout region that contains all of the coding sequence. Knock out the region will result in disruption of protein function. The genotype primers for knockout (KO) mice are 5′-GCATAAAGCCCAGAGCATCACA-3′, 5′-GTTTGGAGTGCTGTTGTGTGTTTG-3′, and the PCR production size is 393 bp. Genotype primers for *Wild-type* (*Wt*) were 5′-CTGGCTTAAAAAGGCTGAACCC-3′, 5′-GCCAGTAGCAAGTAGGGTACATCCTT-3′, and the PCR production size is 348 bp. The schematic knockout and genotyping were shown in Figure S1. Accordingly, all comparisons were made between knockout mice and their *Wt* littermate controls.

### Dextran sulfate sodium–induced colitis

We used 6-week-old *B3gnt8*^*−/−*^ mice (female, n = 6; male, n = 6) and their wild-type (*Wt*) (female, n = 9; male, n = 8) littermates for dextran sulfate sodium (DSS)–induced colitis experiments. The sample sizes were chosen based on established standards in the field for similar phenotypic and biochemical analyses in mouse models, which reliably detect significant effects. Acute colitis was induced by administration of 3% DSS (36–50 kDa; MP Biomedicals) in drinking water for 7 days. In the experiments, the mice were anesthetized with sodium pentobarbital (50 mg/kg, i.p.; #57–33–0; Sigma). All procedures involving mice were performed at Experimental Animal Center of Xinhua Hospital and approved by the Institutional Animal Care and Use Committee of Xinhua Hospital School of Medicine, Shanghai Jiao Tong University (No. XHEC-C-F-2022–010).

### Intestinal characterization

Intestinal tissues of each mouse were fixed in 4% paraformaldehyde (PFA) for 24 h and sectioned at 4 μm for hematoxylin and eosin (H&E) staining. Villus height and crypt depth were measured using NIH Image software with a Nikon microscope. Villus height was assessed from five well-oriented villi per slide, analyzing five fields per section. Goblet cells were counted, and mucous secretions quantified using Alcian blue/periodic acid–Schiff (AB/PAS) staining, calculating goblet cell counts from ten well-oriented villi.

### Histological score

We graded histological changes in the intestinal mucosa as previously described (43, 44). Histological scores were determined blindly based on the sum of epithelial and infiltration scores. Epithelial scores: 0 = normal; 1 = loss of goblet cells in small areas; 2 = loss of goblet cells in large areas; 3 = loss of crypts in small areas; 4 = loss of crypts in large areas. Infiltration scores: 0 = normal; 1 = infiltrate around crypt base; 2 = moderate infiltrate reaching muscularis mucosae; 3 = extensive infiltration reaching muscularis.

### Transmission electron microscopy

We prepared intestinal tissues for transmission electron microscopy (TEM) examination followed protocols (45, 46). Tissues from 6-week-old *B3gnt8*^*−/−*^ mice and their *Wt* littermates were fixed in 2.5% glutaraldehyde at room temperature. After washing, the tissues were postfixed with 1% osmium tetroxide in sodium cacodylate buffer (pH 7.4) at 4 °C for 2 h, stained with saturated uranyl acetate for 3.5 h at room temperature, dehydrated in graded alcohol, and embedded in Eponate 12 resin (Ted Pella, Inc). Sections were cut with a diamond knife and stained with a saturated solution of uranyl acetate in 50% ethanol and lead citrate. We examined and photographed the sections using a Philips CM120 transmission electron microscope (Philips Healthcare) at 80 kV.

### Scanning electron microscopy

We cut ∼5 mm^2^ gut mucosa from *B3gnt8*^*−/−*^ and *Wt* littermate mice and fixed them with 2.5% GLUT overnight at 4 °C. The tissues were rinsed, dehydrated in ethyl alcohol, dried with carbon dioxide, coated with gold, and examined under a Hitachi S-4800 field emission scanning electron microscope (SEM; Hitachi).

### 16S rRNA sequencing

Total microbial genomic DNA was extracted from feces of *B3gnt8*^*−/−*^ (n = 5) and *Wt* mice (n = 5) using a DNeasy PowerSoil Kit (QIAGEN) following the manufacturer's instructions. PCR amplification of the bacterial 16S rRNA gene V4–V5 region was conducted with forward primer 515F (5′-GTGCCAGCMGCCGCGGTAA-3′) and reverse primer 907R (5′-CCGTCAATTCMTTTRAGTTT-3′), incorporating sample-specific 7-bp barcodes for multiplex sequencing. PCR amplicons were purified with Agencourt AMPure Beads (Beckman Coulter) and quantified using a PicoGreen dsDNA Assay Kit (Invitrogen). After quantification, amplicons were pooled equally and subjected to paired-end (PE) 2 × 300 bp sequencing on an Illumina MiSeq platform with MiSeq Reagent Kit version three at Shanghai Personal Biotechnology Co., Ltd. Sequencing data were processed using the QIIME pipeline (version 1.8.0; https://qiime.org), as previously described. Sequence analysis primarily utilized QIIME and R software version 3.2.0 (R Foundation for Statistical Computing).

### RNA sequencing

RNA-Seq (RNA Sequencing) was conducted by Sangon Biotech. Total RNA was extracted from the small intestinal and colonic mucosa of *B3gnt8*^*−/−*^ mice (n = 3–5) and *Wt* mice (n = 3–5), including samples from the proximal small intestine, distal small intestine, and colon; each group had five biological replicates. The extraction used the Total RNA Extractor kit (B511311), following the manufacturers’ protocol. Sequencing libraries were constructed with the VAHTSTM mRNA-seq V2 Library Prep Kit according to manufacturer guidelines. Library quality was assessed using the Agilent Bioanalyzer 2100 system. Paired-end sequencing was performed on NovaSeq sequencers (Illumina). FastQC (version 0.11.2) evaluated data integrity. Clean reads were aligned to a reference genome using HISAT2 (version 2.0) with default parameters. For statistical analysis of alignment results, RSeQC (version 2.6.1) was utilized effectively. Gene expression values for transcripts were calculated using StringTie (version 1.3.3 b). Principal Component Analysis (PCA) and Principal Coordinates Analysis (PCoA) were performed to assess sample distances and differences among groups. Differentially expressed genes (DEGs) between two samples were identified through DESeq2 (version 1 12.4). Functional enrichment analyses—including Gene Ontology (GO) assessments and Kyoto Encyclopedia of Genes and Genomes (KEGG)—were conducted to identify significant enrichments in GO terms or metabolic pathways related to DEGs.

### Western blotting

For Western blotting (WB), we homogenized ∼50 mg tissue in 500 μl RIPA buffer (Invitrogen) with a protease inhibitor cocktail (Servicebio). Protein concentration was determined using BCA reagent (Pierce Biotechnology [Thermo Fisher]). Equal amounts of protein were separated on 10% NuPAGE Bis-Tris gels (Invitrogen) and transferred to PVDF membranes (MilliporeSigma). After blocking with 5% nonfat milk, membranes were incubated overnight at 4 °C with primary antibodies listed in Table S1. Membranes were washed three times with TBST and then incubated with secondary antibodies. Following final washes, signals were detected using an ECL Reagent Kit (Pierce).

### Immunofluorescence (IF) and immunochemistry (IHC)

Immunofluorescence (IF) and immunochemistry (IHC) assays were performed as we described previously (47). The intestinal tissues were fixed in 4% paraformaldehyde for 24 h, followed by dehydration, clearing, and paraffin embedding. Sections were cut at 4 μm thick and mounted on positively charged slides, then incubated with xylol and decreasing concentrations of ethanol. After antigen retrieval, blocking was done with 5% bovine serum albumin for 30 min at room temperature. Primary antibodies were incubated overnight at 4 °C in a humid chamber. The next day, slides were washed with phosphate-buffered saline (PBS) and incubated with the secondary antibody for 50 min at room temperature away from light. Details of the primary antibodies used are provided in Table S1. Image J software (National Institutes of Health, NIH, http://rsbweb.nih.gov/nih-image/) was used to quantify the results of IHC and IF assays. The Average Optical Density (AOD) was used to IHC using 5-field from one sample and got averaged AOD. The Mean Integrated Density (MID) was used to IF using 5-field from one sample and got averaged MID to graph.

### Statistics and reproducibility

Numerical source data for all charts are provided in Methods and Figure legends. Statistical tests were conducted using GraphPad Prism 10 Software (GraphPad), employing two-tailed unpaired t-tests for two groups and one-way ANOVA for multiple comparisons. Each mouse was treated as an individual sample. Data were obtained from at least three independent experiments, with representative results shown as mean ± standard error of the mean (SEM). *p* values < 0.05 were deemed statistically significant, with significance levels further categorized as ∗*p* < 0.1, ∗∗*p* < 0.01, ∗∗∗*p* < 0.001, ∗∗∗∗*p* < 0.0001.

### Data availability

The data generated or analyzed during this study are available from the corresponding author upon reasonable request. Source images for representative Western blots and Immunofluorescence Staining shown in figures are provided in Figures S11 and S12. The datasets about RNA-sequencing and 16S rRNA-sequencing were stored in Figshare (https://figshare.com/s/ad13d8c21cdb56ab1b50).

## Supporting information

This article contains supporting information.

## Conflict of interest

The authors declare that they do not have any conflicts of interest with the content of this article.
