# Supplementary material for: B3GNT8-mediated glycosylation maintains intestinal homeostasis and protects against colitis
Source: J Biol Chem. 2025 Dec 10;302(2):111014. doi: 10.1016/j.jbc.2025.111014 (PMC12805103; doi:10.1016/j.jbc.2025.111014)
Supplement: Supplementary data [file mmc1.pdf]

Supplementary information

B3GNT8-Mediated Glycosylation Maintains Intestinal Homeostasis and Protects Against Colitis

Haoyun Mao , Yi Cao, Ying Lu, Shicheng Peng, Bo Wu, and Ying Wang, Yongtao Xiao

Supplemental Information contents:

Supplementary Table 1, Page 2 - 3

Supplementary Figures 1-12, Page 4 – Page 37

Supplementary Table 1 Antibody information

Antibody	Source	Catalog#	Application/dilution
Anti-β-actin	Santa cruze	SC-47778	WB (1:2000)
Anti-B3GNT8	Boster	PB0725	WB (1:1000)<br>IF(1:200)/ IHC(1:200)
Anti-Lysozyme	Servicebio	GB11345	WB (1:1000)<br>IF(1:2000)
Anti-LAMP1	DSHB	G1/139-5-S	WB (1:1000)
Anti-LAMP2	DSHB	ABL-93-S	WB (1:1000)<br>IF(1:200)
Anti-LC3A/B	Cell Signaling Technology ,CST	12741S	WB (1:1000)<br>IF(1:200)
Anti-NLRP3	Abclonal	A5652	WB (1:2000)
Anti-RORγt	Bioss	bs-23110R	WB (1:1000)
Anti-Il-1β	Santa cruze	SC-12742	WB (1:2000)
Anti-E-Cadherin	BD Biosciences	560061	IF (1:200)
Anti-WGA	Servicebio	G1731	IF (1:200)
Anti-ZO-1	Proteintech	82870-7-RR/PRO	WB (1:1000)<br>IF(1:500)
Anti-Occludin	Abnova	H00004950-M01	WB (1:2000)
Anti-Claudin-1	Cell Signaling Technology ,CST	#13255	WB (1:1000)
Anti-Ki-67	Servicebio	GB111141	IHC(1:500)
Anti-ATG16L1	Cell Signaling Technology ,CST	#8089P	WB (1:1000)
Anti-ATG12	Proteintech	30505-1-AP	WB (1:1000)
Anti-P-STAT3	Cell Signaling Technology ,CST	#91455	WB (1:1000)
Anti-STAT3	Cell Signaling Technology ,CST	#9139P	WB (1:1000)

Anti-MPO	Servicebio	GB120016	IHC (1:250)
Anti-TBX21	Bioss	bs-3599R	IHC (1:200)
Anti-CD68	Servicebio	GB115723	IHC (1:250)
Anti-MUC2	Servicebio	GB11344	IF (1:500)
Anti-B3GNT2	Boster	M09524	IF(1:100)

Supplementary Figure 1: B3GNT8 is enriched in gastroenterological tract.

(A) UDP-GlcNAc:betaGal beta-1,3-N-acetylglucosaminyltransferase 8 (B3GNT8) mRNA is specifically expressed in Esophagus, Intestine and Vagina (Human Protein Atlas proteinatlas.org).

(B) Representative immunohistochemistry (IHC) images depicting the localization of B3GNT8 in the human intestinal epithelium (Human Protein Atlas proteinatlas.org).

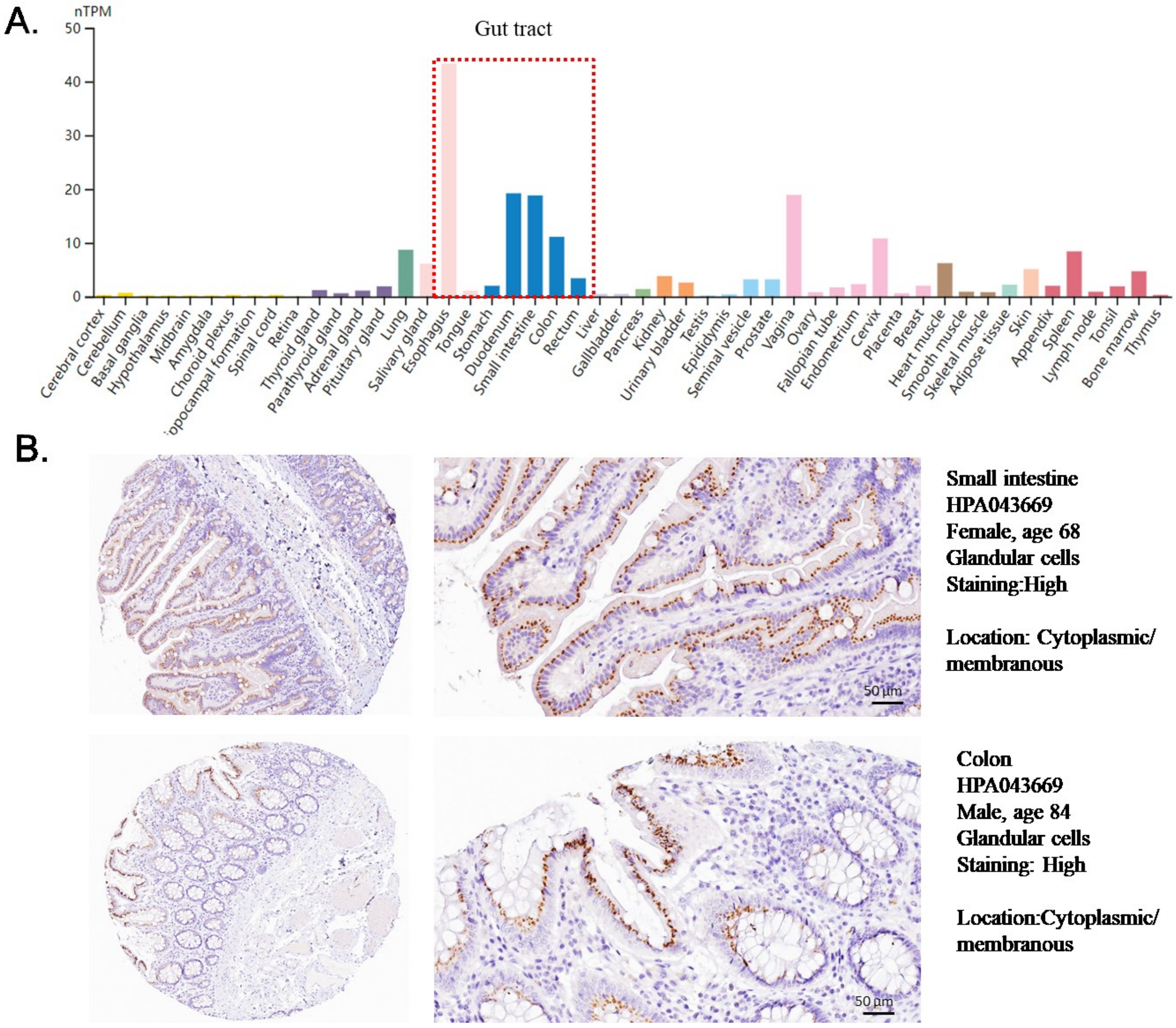

Supplementary Figure 2. The alterations of B3GNT8 in intestines of human and mice.

(A) Representative immunohistochemistry (IHC) images of B3GNT8 localization in the normal intestinal tissues from pediatric patients diagnosed intestinal failure.

(B) (B) Alteration of *B3gnt8* mRNA and *Ccnd1* mRNA from the embryonic stages (E12.5 – 17.5) to the postnatal time (P0 – 13.5) (each group, n = 4).

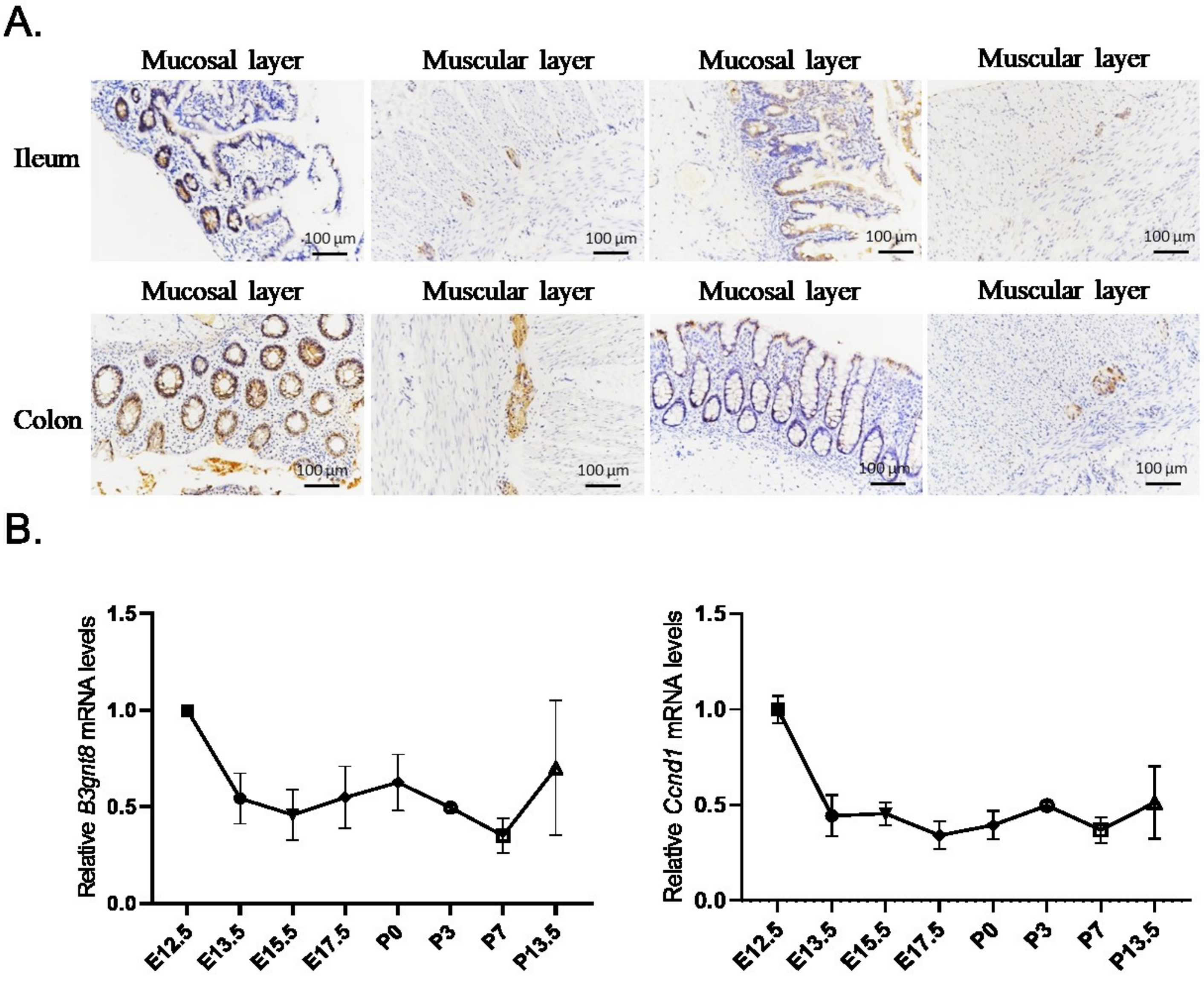

Supplementary Figure 3 B3GNT8 is enriched intestinal Paneth cells and goblet cells. RNA single cell type specificity showed B3GNT8 was specially enriched in Paneth cells and goblet cells (Human Protein Atlas proteinatlas.org).

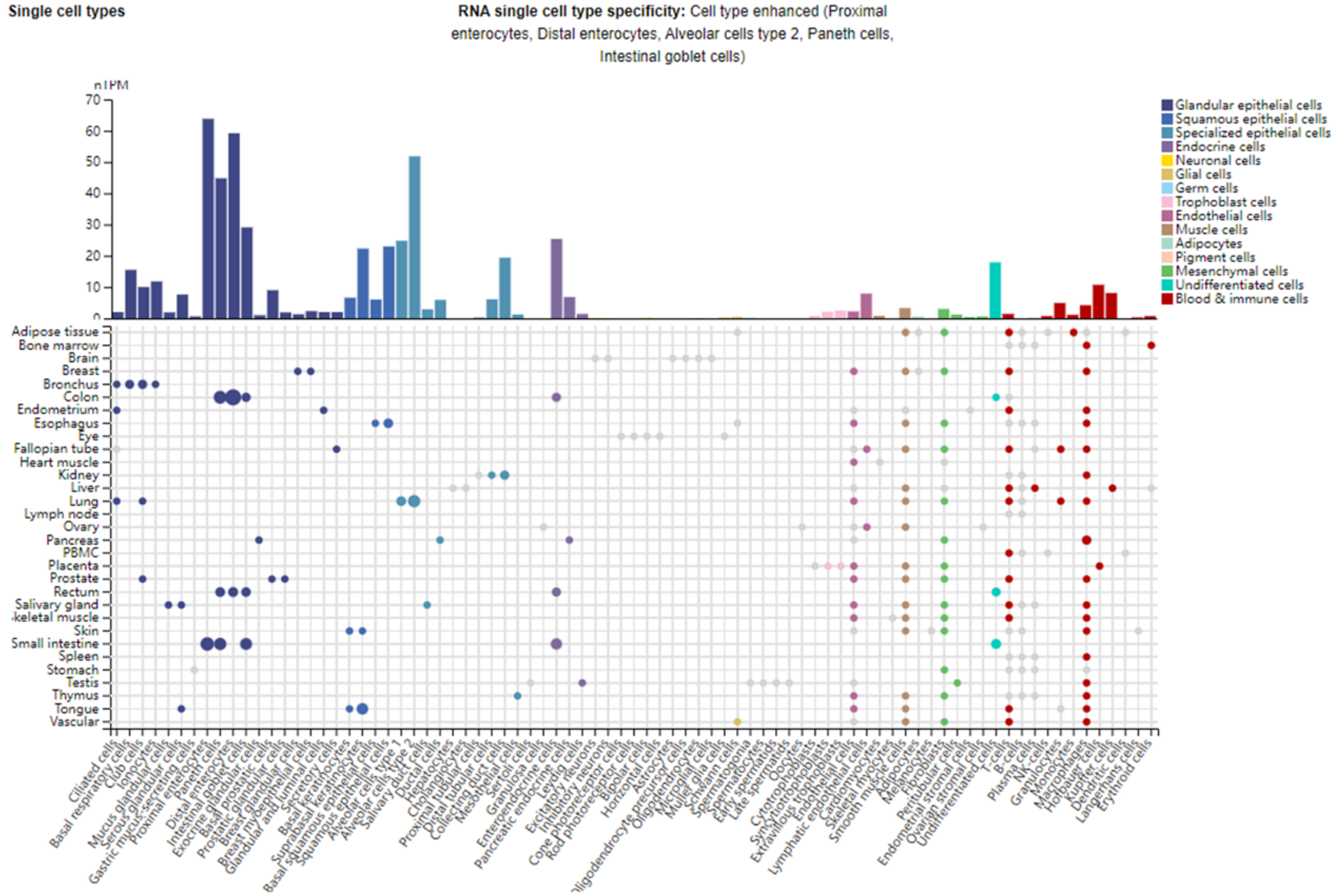

Supplementary Figure 4 Immunofluorescence analysis of B3GNT2 and B3GNT8 expression in pediatric inflammatory bowel disease (IBD).

(A) Representative images of immunofluorescence (IF) co-staining between B3GNT8 and B3GNT2, B3GNT2 and Lysozyme, and B3GNT2 and Mucin 2 (MUC2) in ileal mucosa from pediatric patients with Crohn's disease (CD, n = 4).

(B) Representative images of immunofluorescence (IF) co-staining between B3GNT8 and B3GNT2, B3GNT2 and Lysozyme, and B3GNT2 and Mucin 2 (MUC2) in colonic mucosa from pediatric patients with ulcerative colitis (UC, n = 4). Representative images from n=4 independent individuals per group are shown. Each data point in the corresponding graphs represents one individual.

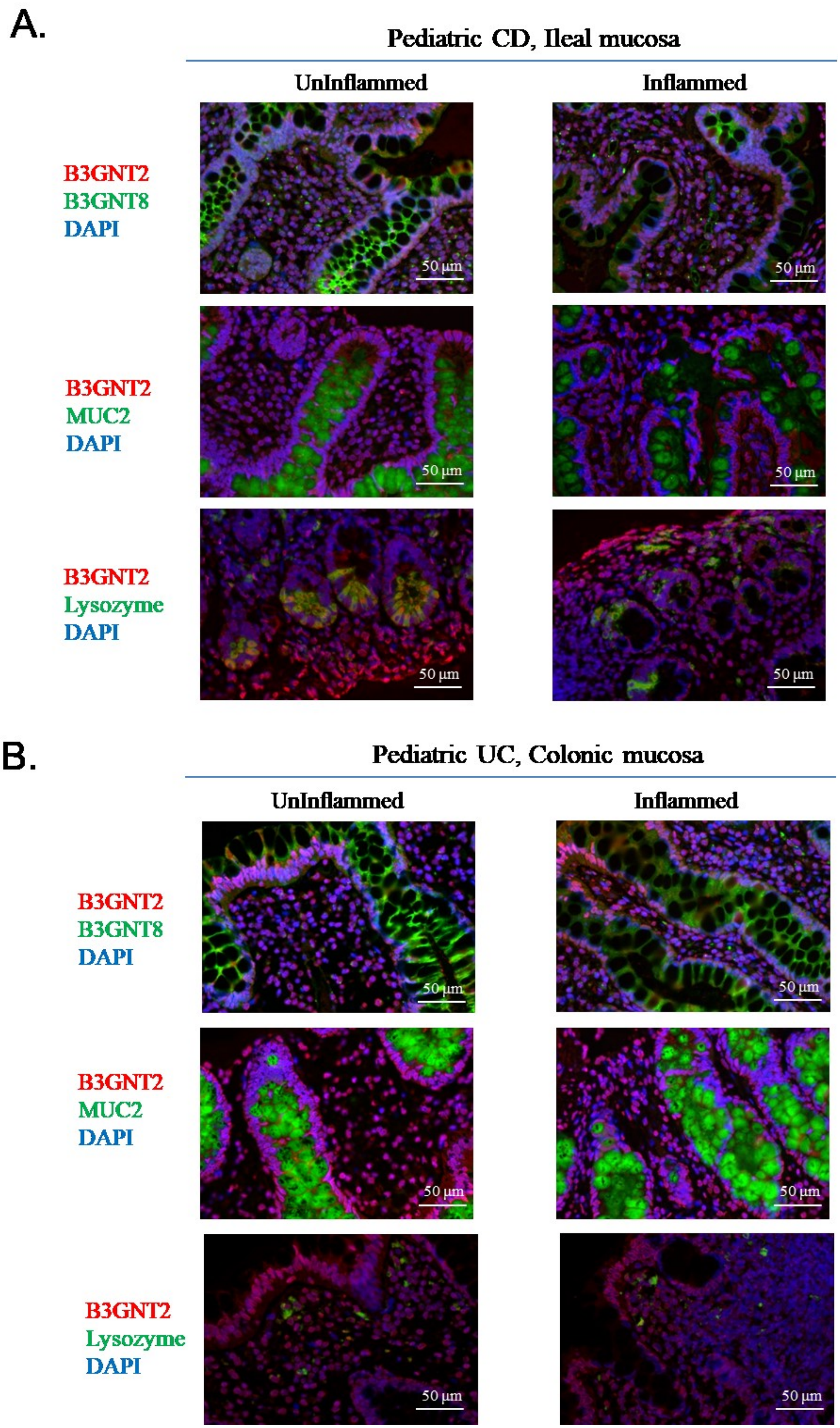

A.
Pediatric CD, Ileal mucosa
UnInflammed
Inflammed
B3GNT2
B3GNT8
DAPI
B3GNT2
MUC2
DAPI
B3GNT2
Lysozyme
DAPI
50 µm
B.
Pediatric UC, Colonic mucosa
UnInflammed
Inflammed
B3GNT2
B3GNT8
DAPI
B3GNT2
MUC2
DAPI
B3GNT2
Lysozyme
DAPI
50 µm

Supplementary Figure 5 Generation of *B3gnt8* knockout (*B3gnt8*$^{-/-}$) mice and genotyping.

(A) The structure of *B3gnt8* gene, exons 1 - 2 of *B3gnt* (ENSMUST00000076034.7) transcript is the knockout region. Knock out the region will result in disruption of protein function. The CRISPR/Cas9 technology to modify *B3gnt8* gene.

(B) Reprehensive image of *B3gnt8* knockout mice genotype using the PCR analysis.

(C) Representative images of western blotting (WB) analysis for *B3gnt8* in small intestines from both *B3gnt8* $^{-/-}$ mice and *Wt* mice (each group, n = 3).

(D) Gross morphological comparison and weight. Statistical significance: Unpaired two-tailed Student's *t* test with or without Welch's correction analysis for (**D**). *** p < 0.001.

(E) Representative images of histology for liver, lung, brain，spleen，kidney and pancrea both *B3gnt8* $^{-/-}$ mice and *Wt* mice (each group, n = 5). *KO*, knockout, *Wt*, wild type; -/-, *B3gnt8* $^{-/-}$; -/+, *B3gnt8* $^{-/+}$; +/+, *Wt*

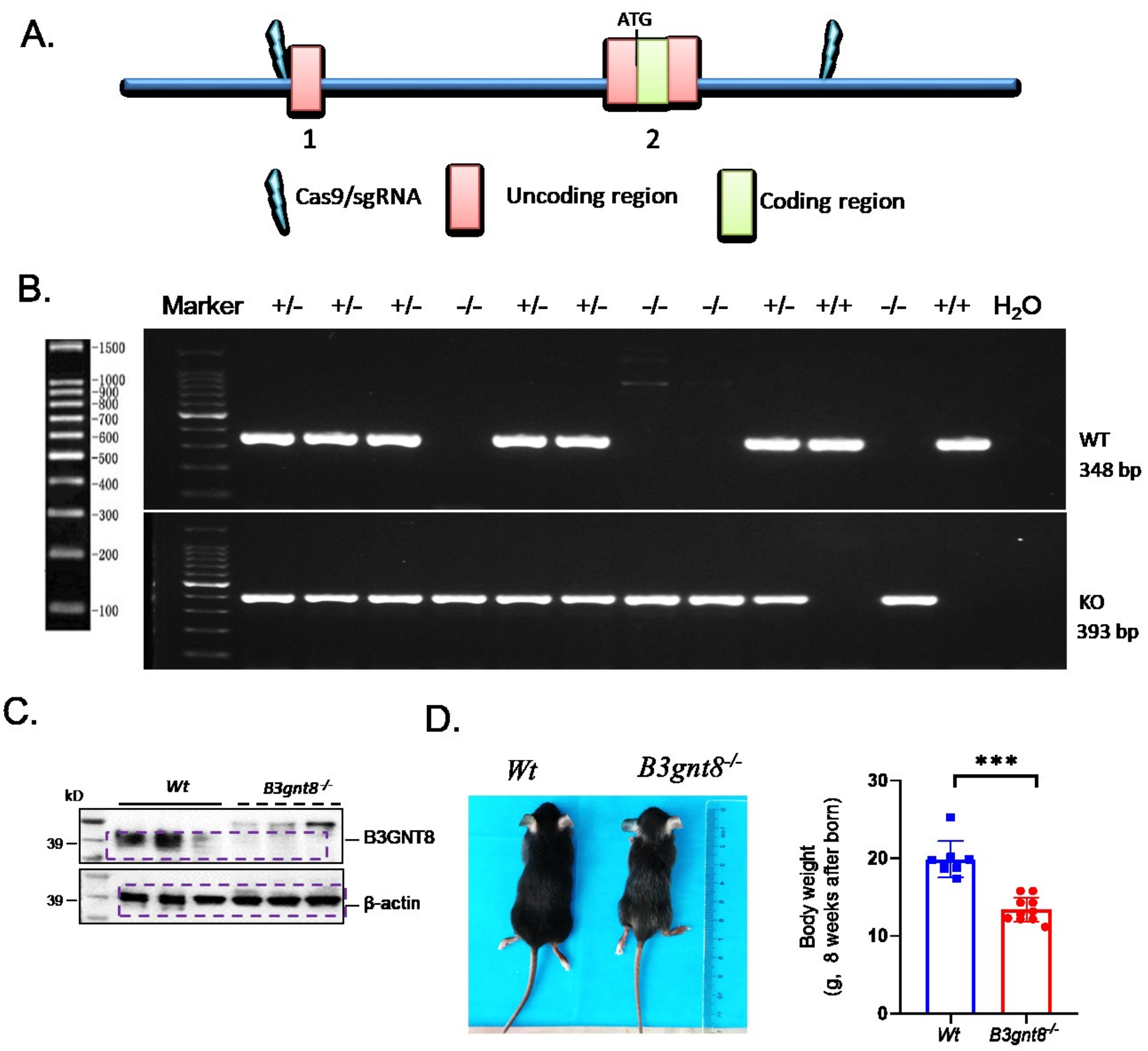
A.
ATG
1
2
Cas9/sgRNA
Uncoding region
Coding region
B.
Marker
+/- +/- +/- -/- +/- +/- -/- -/- +/- +/+ -/- +/+ H2O
1500
1000
900
800
700
600
500
400
300
200
100
WT
348 bp
KO
393 bp
C.
kD
Wt
B3gnt8-/-
39
B3GNT8
39
β-actin
D.
Wt
B3gnt8-/-
***
Body weight
(g, 8 weeks after born)
30
20
10
0
Wt
B3gnt8-/-

E.

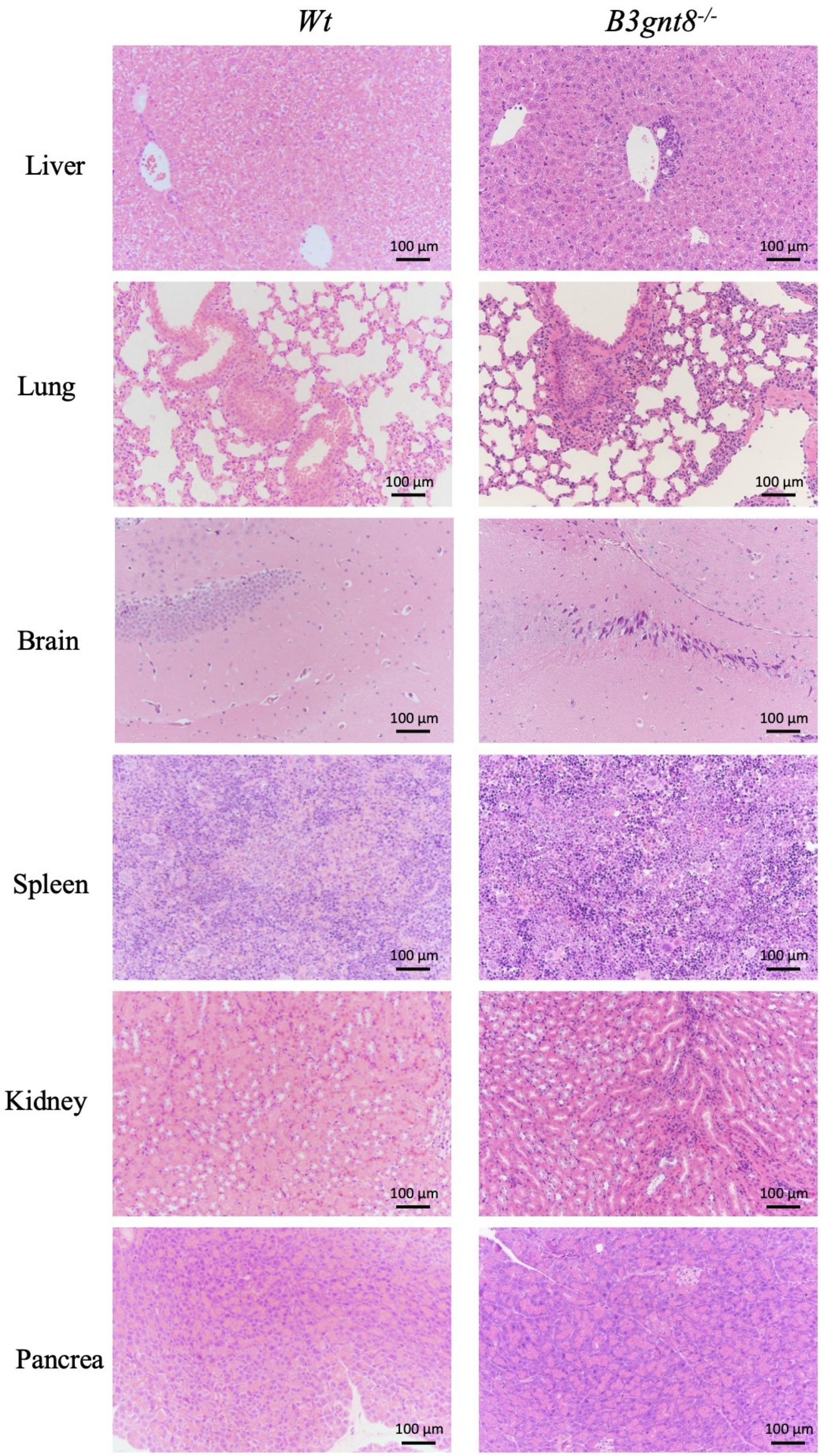

Supplementary Figure 6 Histological alterations in intestines between *B3gnt8* knockout (*B3gnt8* $^{-/-}$) and wild type (*Wt*) mice.

(A) Comparative gross anatomy of the intestines from *Wt* and *B3gnt8* $^{-/-}$ mice.

(B) Quantification of colon and small intestine length in *Wt* and *B3gnt8* $^{-/-}$ mice.

(C) Representative images of histology for proximal (pro), middle (mid), distal (dis) small bowel and colon from both *B3gnt8* $^{-/-}$ mice and *Wt* mice (each group, n = 5-7).

(D) Quantification of histological scores in panel (**C**). Statistical significance: Unpaired two-tailed Student's t test with or without Welch's correction analysis for (**B**) and (**D**). ns, not significant, * $p < 0.05$, ** $p < 0.01$

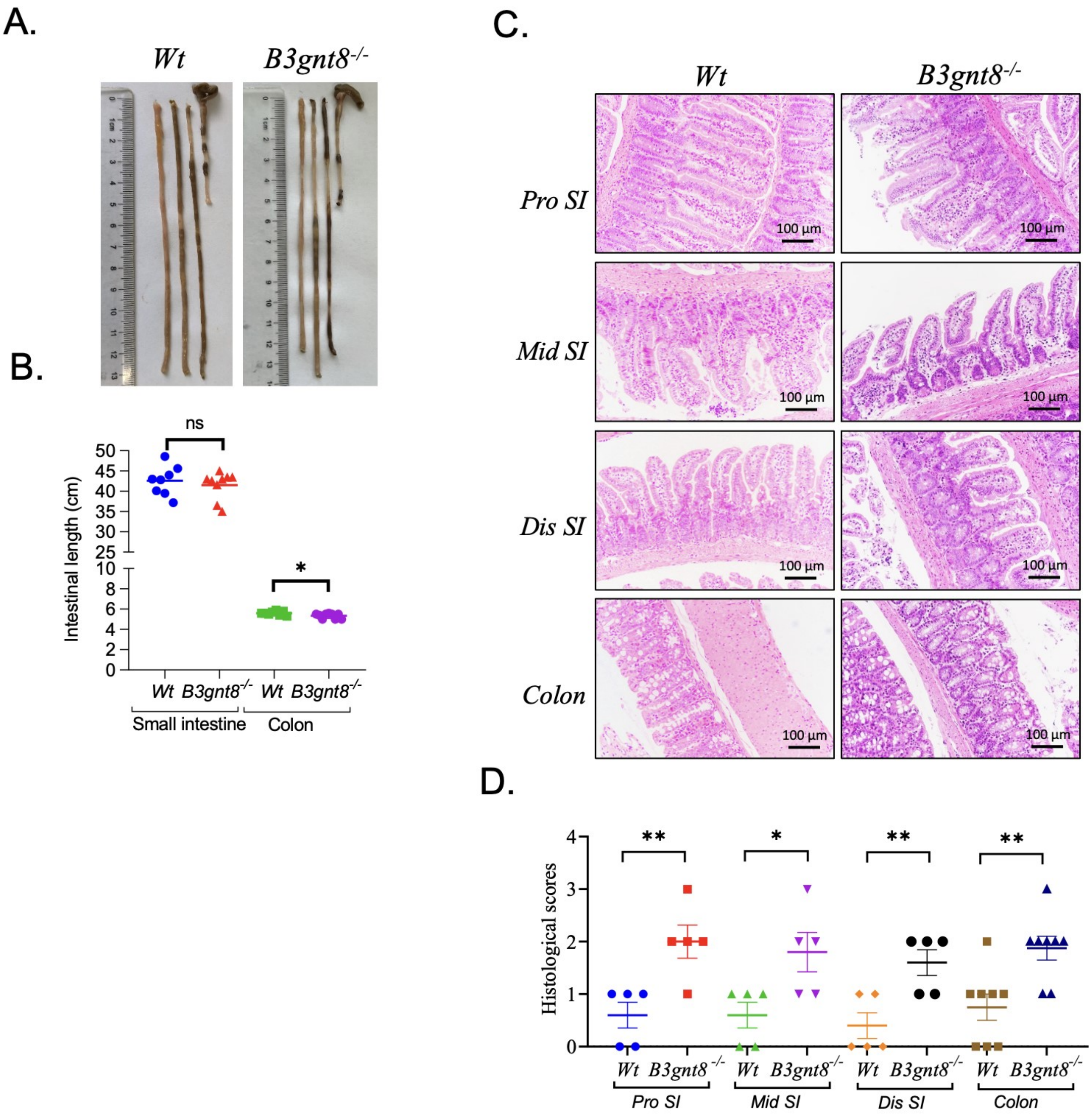
A.
Wt
B3gnt8-/-
B.
ns
*
Intestinal length (cm)
50
45
40
35
30
25
10
8
6
4
2
0
Wt
B3gnt8-/-
Wt
B3gnt8-/-
Small intestine
Colon
C.
Wt
B3gnt8-/-
Pro SI
Mid SI
Dis SI
Colon
100 µm
D.
**
*
**
**
Histological scores
4
3
2
1
0
Wt
B3gnt8-/-
Wt
B3gnt8-/-
Wt
B3gnt8-/-
Wt
B3gnt8-/-
Pro SI
Mid SI
Dis SI
Colon

Supplementary Figure 7 *B3gnt8* deficiency altered the microbiota composition in feces.

(A) Principal co-ordinates analysis (PcoA for altered bacteria in the feces of *B3gnt8* knockout (*B3gnt8*$^{-/-}$) and wild type (*Wt*) mice (Each group, n = 5).

(B) The relative abundance of the top bacteria (phylum) in the feces of *B3gnt8*$^{-/-}$ mice and *Wt* mice. WT, wild type; B3gnt8-F, *B3gnt8*$^{-/-}$ feces.

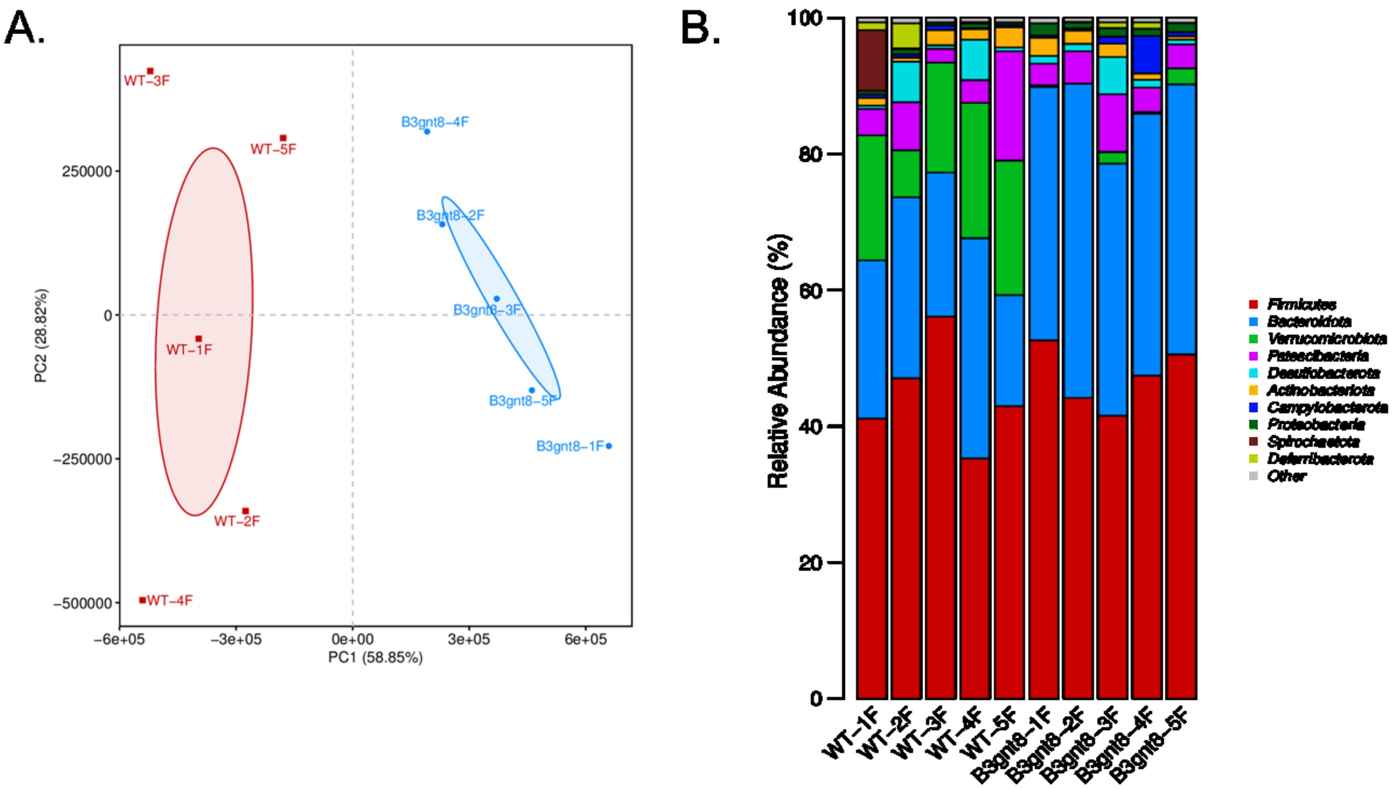

Supplementary Figure 8 *B3gnt8* deficiency altered immune response in small intestines.

(A) Representative image of Ki-67, TBX21, RORγt and CD68 immunochemistry stain (IHC) in distal (Dis) small intestines from both *B3gnt8*$^{-/-}$ (n = 5) mice and *Wt* (n = 5) mice.

(B) Quantification of them in panel (**A**). Statistical significance: Unpaired two-tailed Student's *t* test with or without Welch's correction analysis for (**B**). ns, not significant, * $p < 0.05$, ** $p < 0.01$, *** $p < 0.001$.

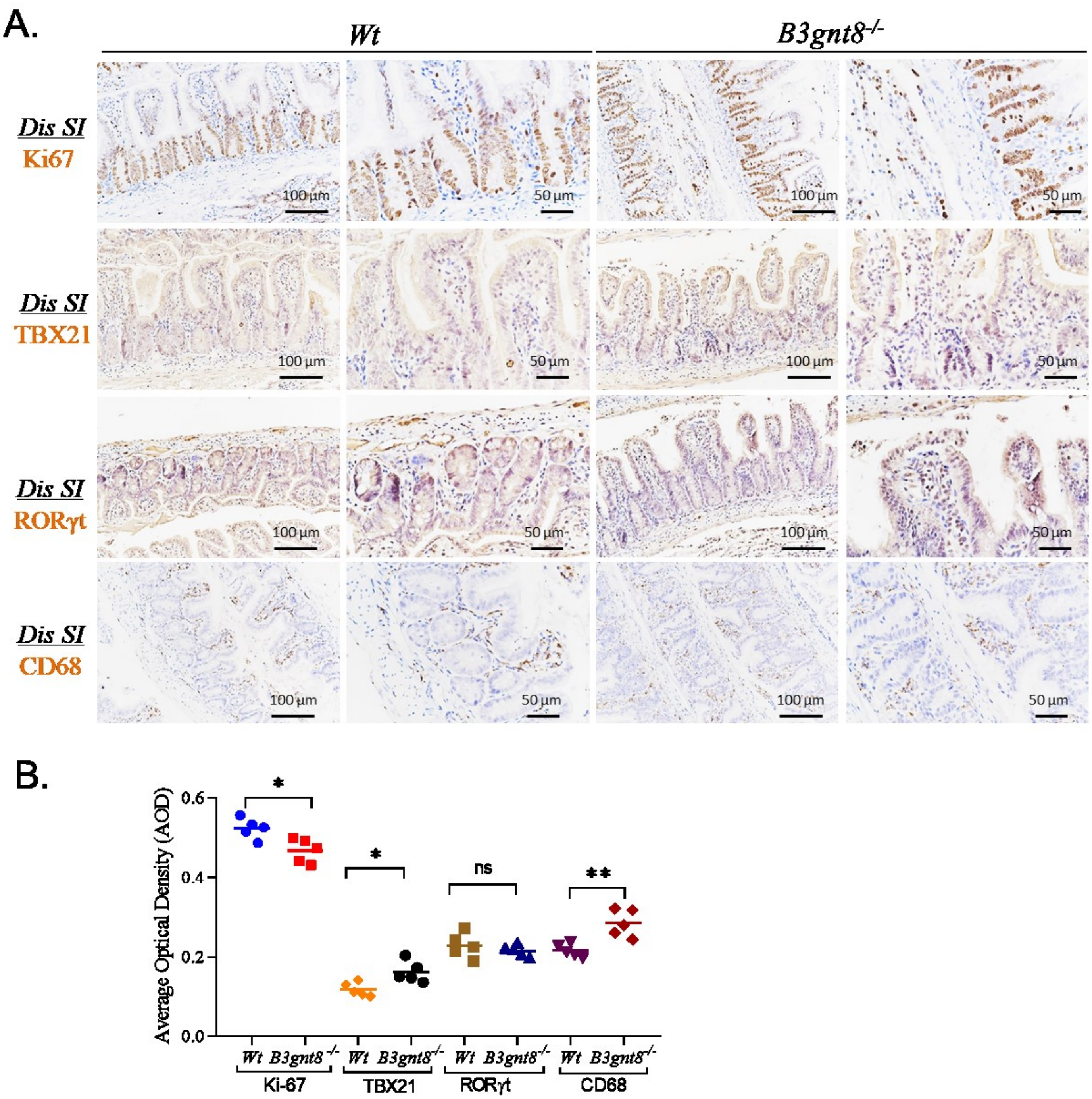
A.
Wt
B3gnt8-/-
Dis SI
Ki67
Dis SI
TBX21
Dis SI
RORγt
Dis SI
CD68
100 µm
50 µm
B.
Average Optical Density (AOD)
0.6
0.4
0.2
0.0
*
*
ns
**
Wt B3gnt8-/-
Ki-67
TBX21
RORγt
CD68

Supplementary Figure 9 The differentially expressed genes in the colonic mucosa of *B3gnt8*$^{-/-}$ mice and *Wt* mice.

(A) Heatmap of differentially expressed genes in colonic mucosa from *B3gnt8* knockout (*B3gnt8*$^{-/-}$) mice (n = 4) and wild type (*Wt*) mice (n = 5).

(B) Gene Ontology (GO) enrichment analysis results of differentially expressed genes in the small intestines of *B3gnt8*$^{-/-}$ mice and *Wt* mice.

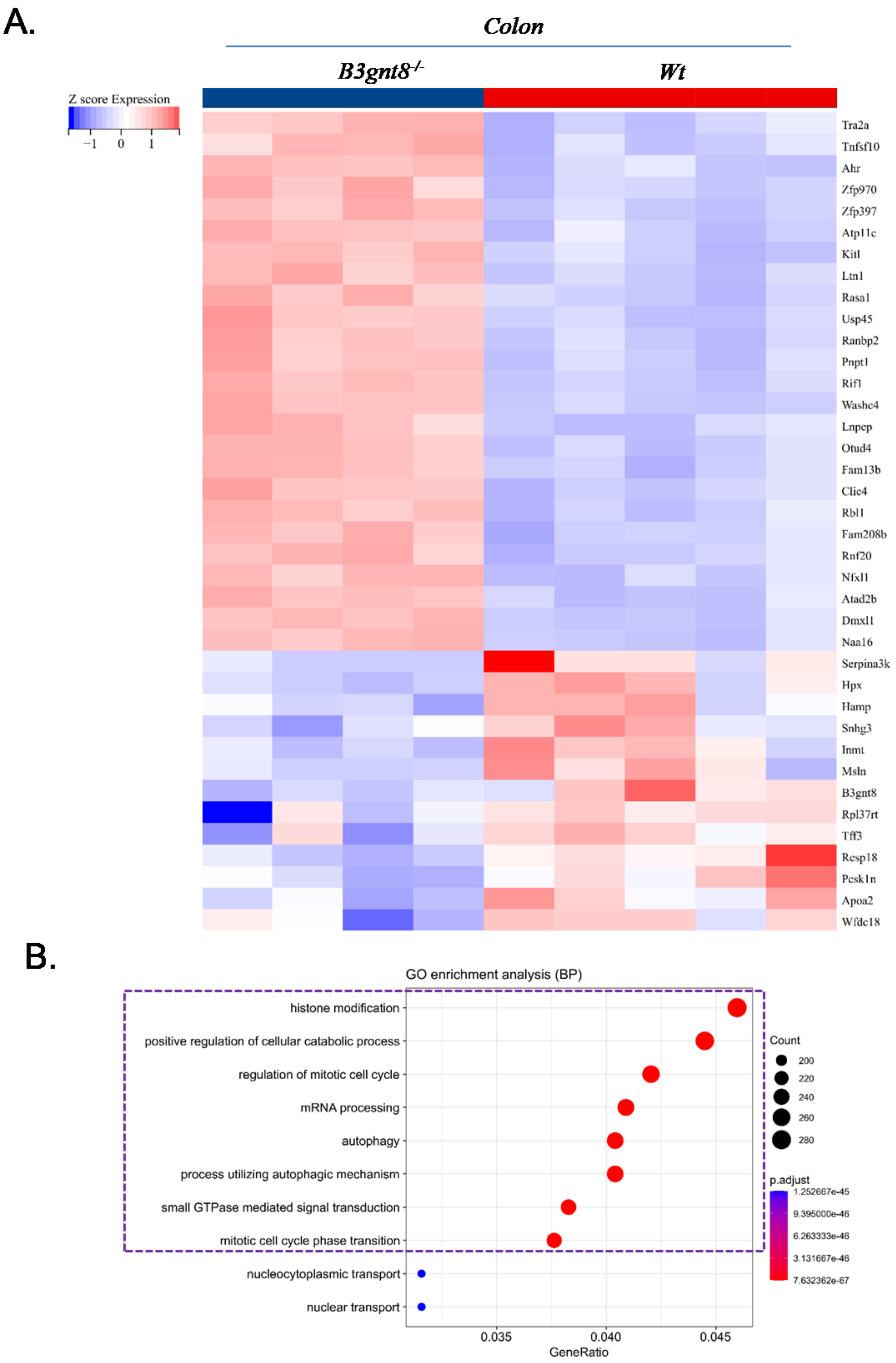
A.
Colon
B3gnt8-/-
Wt
Z score Expression
−1 0 1
Tra2a
Tnfsf10
Ahr
Zfp970
Zfp397
Atp11c
Kitl
Ltn1
Rasa1
Usp45
Ranbp2
Pnpt1
Rif1
Washc4
Lnpep
Otud4
Fam13b
Clic4
Rbl1
Fam208b
Rnf20
Nfxl1
Atad2b
Dmxl1
Naa16
Serpina3k
Hpx
Hamp
Snhg3
Inmt
Msln
B3gnt8
Rpl37rt
Tff3
Resp18
Pcsk1n
Apoa2
Wfdc18
B.
GO enrichment analysis (BP)
histone modification
positive regulation of cellular catabolic process
regulation of mitotic cell cycle
mRNA processing
autophagy
process utilizing autophagic mechanism
small GTPase mediated signal transduction
mitotic cell cycle phase transition
nucleocytoplasmic transport
nuclear transport
0.035
0.040
0.045
GeneRatio
Count
200
220
240
260
280
p.adjust
1.252667e-45
9.395000e-46
6.263333e-46
3.131667e-46
7.632362e-67

Supplementary Figure 10 Aggravated colitis susceptibility in *B3gnt8* knockout (*B3gnt8* $^{-/-}$) mice upon DSS challenge.

(A) Body weight changes in *B3gnt8* $^{-/-}$ mice and *Wt* mice (each group, n = 5).

(B) Representative images of gross intestinal pathology from *Wt* and *B3gnt8* $^{-/-}$ mice following DSS challenge.

(C) Quantification of colon length in *Wt* and *B3gnt8* $^{-/-}$ mice (**B**).

(D) Representative images of histology for proximal (pro), middle (mid), distal (dis) small bowel from both *B3gnt8* $^{-/-}$ mice and *Wt* mice following DSS challenge (each group, n = 5).

(E) Quantification of histological scores in panel (**D**). Statistical significance: Unpaired two-tailed Student's t test with or without Welch's correction analysis for (**C**) and (**E**). ns, not significant, * $p < 0.05$, ** $p < 0.01$.

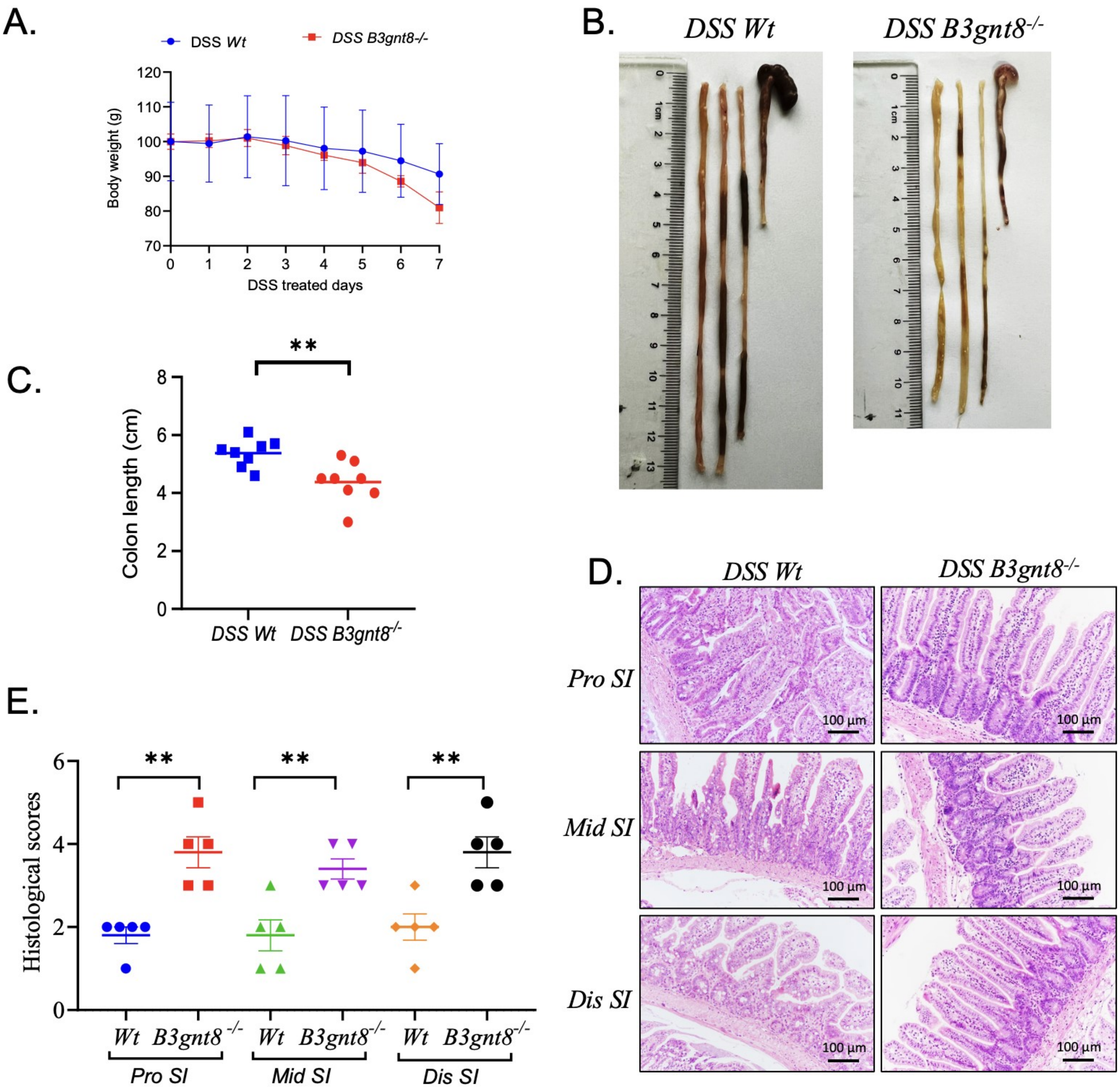
A.
DSS Wt
DSS B3gnt8-/-
Body weight (g)
DSS treated days
B.
DSS Wt
DSS B3gnt8-/-
C.
Colon length (cm)
**
DSS Wt
DSS B3gnt8-/-
D.
DSS Wt
DSS B3gnt8-/-
Pro SI
Mid SI
Dis SI
100 µm
E.
Histological scores
**
Wt
B3gnt8 -/-
Pro SI
Mid SI
Dis SI

Supplementary Figure 11 Original WB bands in Figures

Figure S4

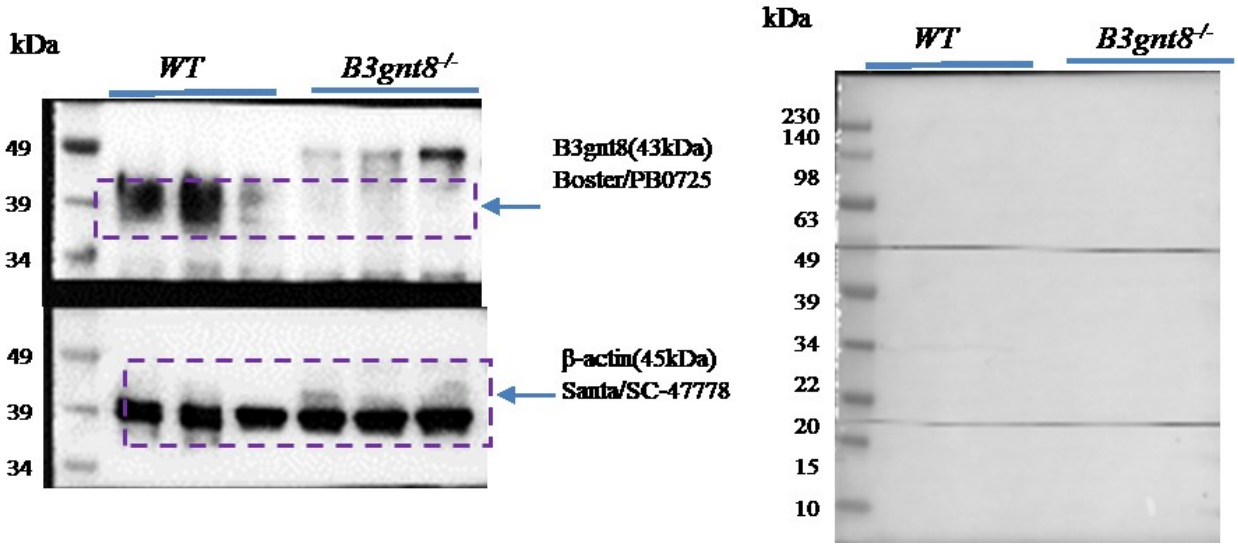

Figure 5

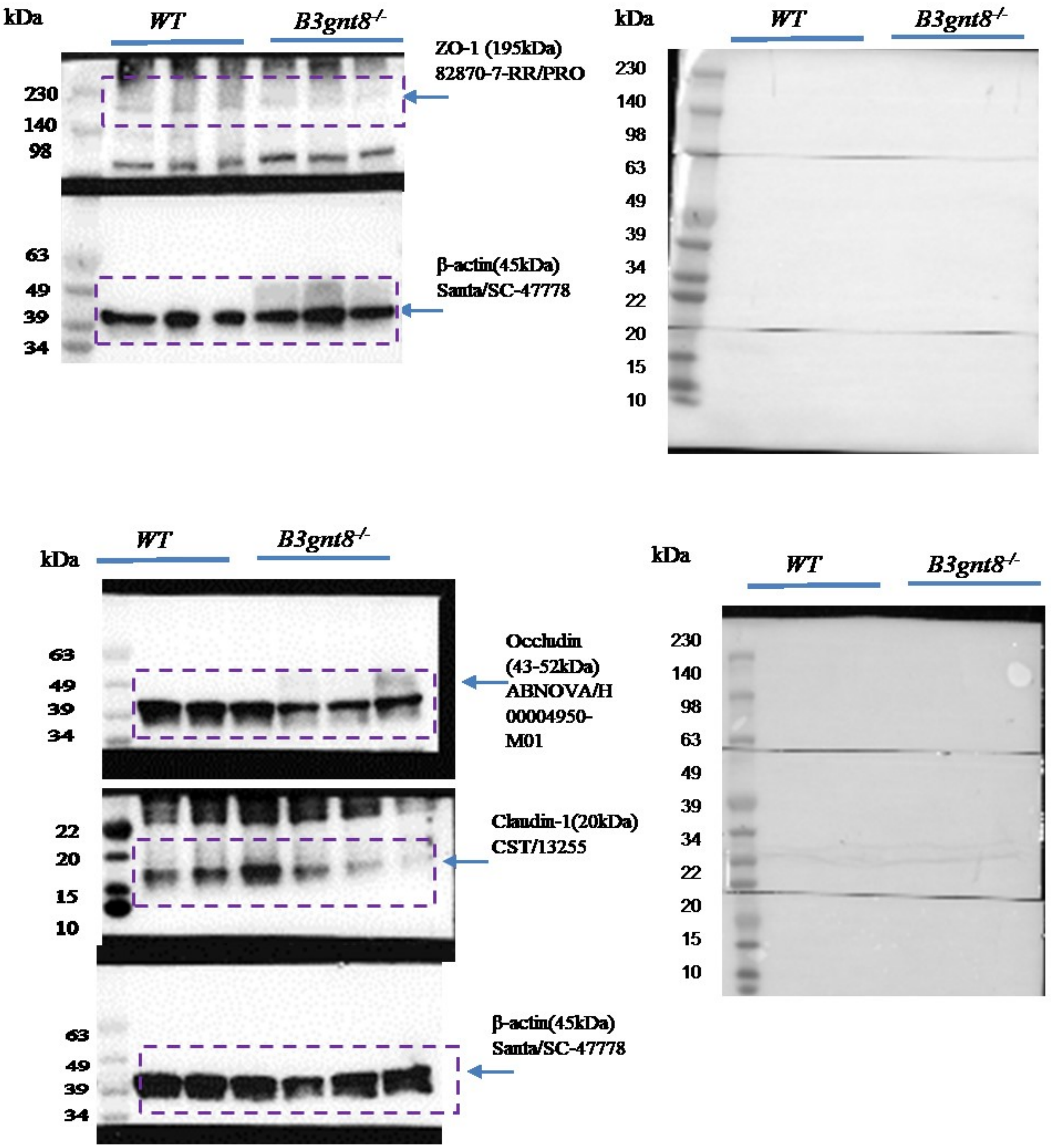
kDa
WT
B3gnt8-/-
230
140
98
63
49
39
34
ZO-1 (195kDa)
82870-7-RR/PRO
β-actin(45kDa)
Santa/SC-47778
22
20
15
10
Occludin
(43-52kDa)
ABNOVA/H
00004950-
M01
Claudin-1(20kDa)
CST/13255
β-actin(45kDa)
Santa/SC-47778

Figure 6

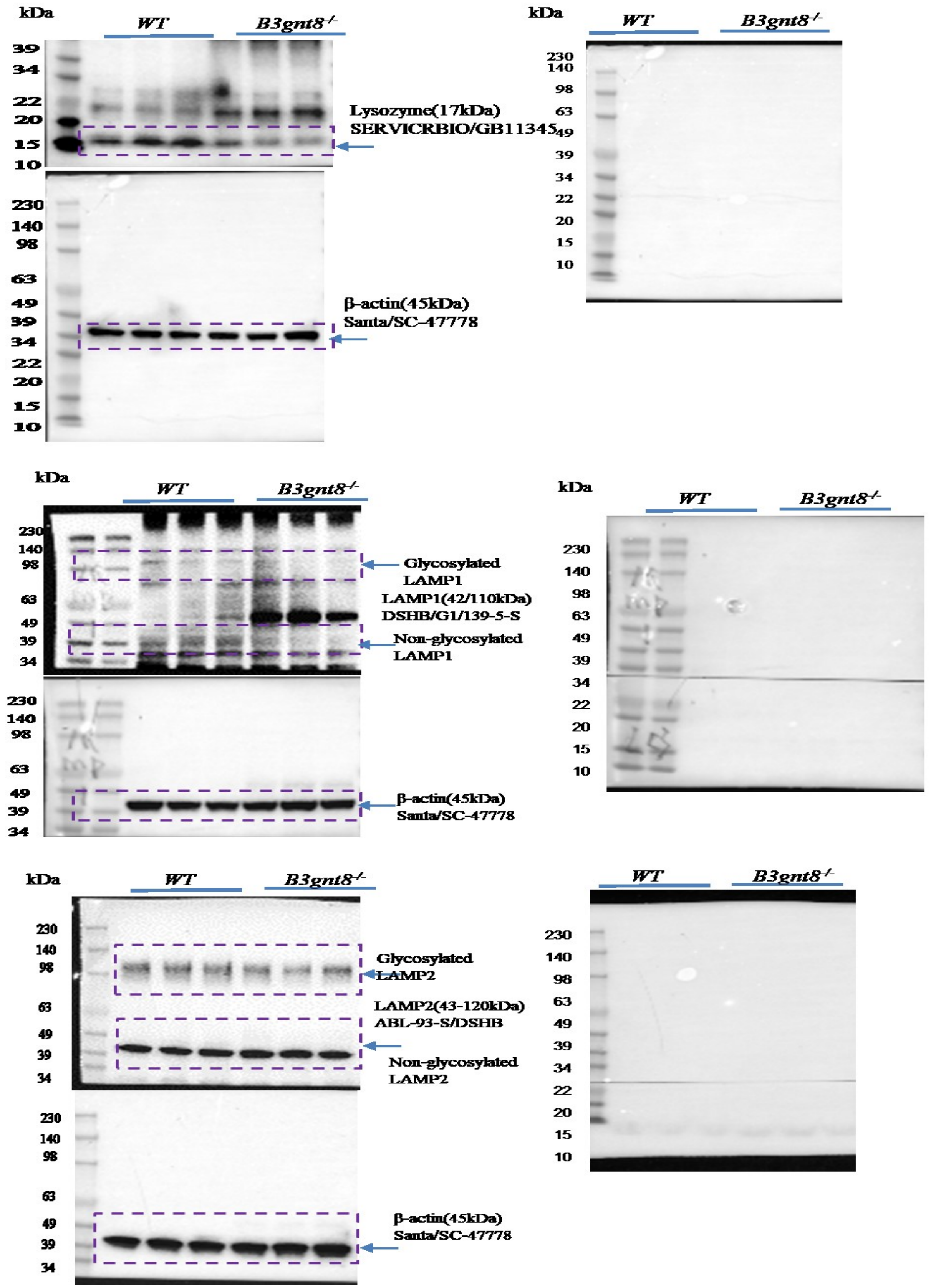
kDa
WT
B3gnt8-/-
Lysozyme(17kDa)
SERVICRBIO/GB11345
β-actin(45kDa)
Santa/SC-47778
Glycosylated LAMP1
LAMP1(42/110kDa)
DSHB/G1/139-5-S
Non-glycosylated LAMP1
β-actin(45kDa)
Santa/SC-47778
Glycosylated LAMP2
LAMP2(43-120kDa)
ABL-93-S/DSHB
Non-glycosylated LAMP2
β-actin(45kDa)
Santa/SC-47778

Figure 8

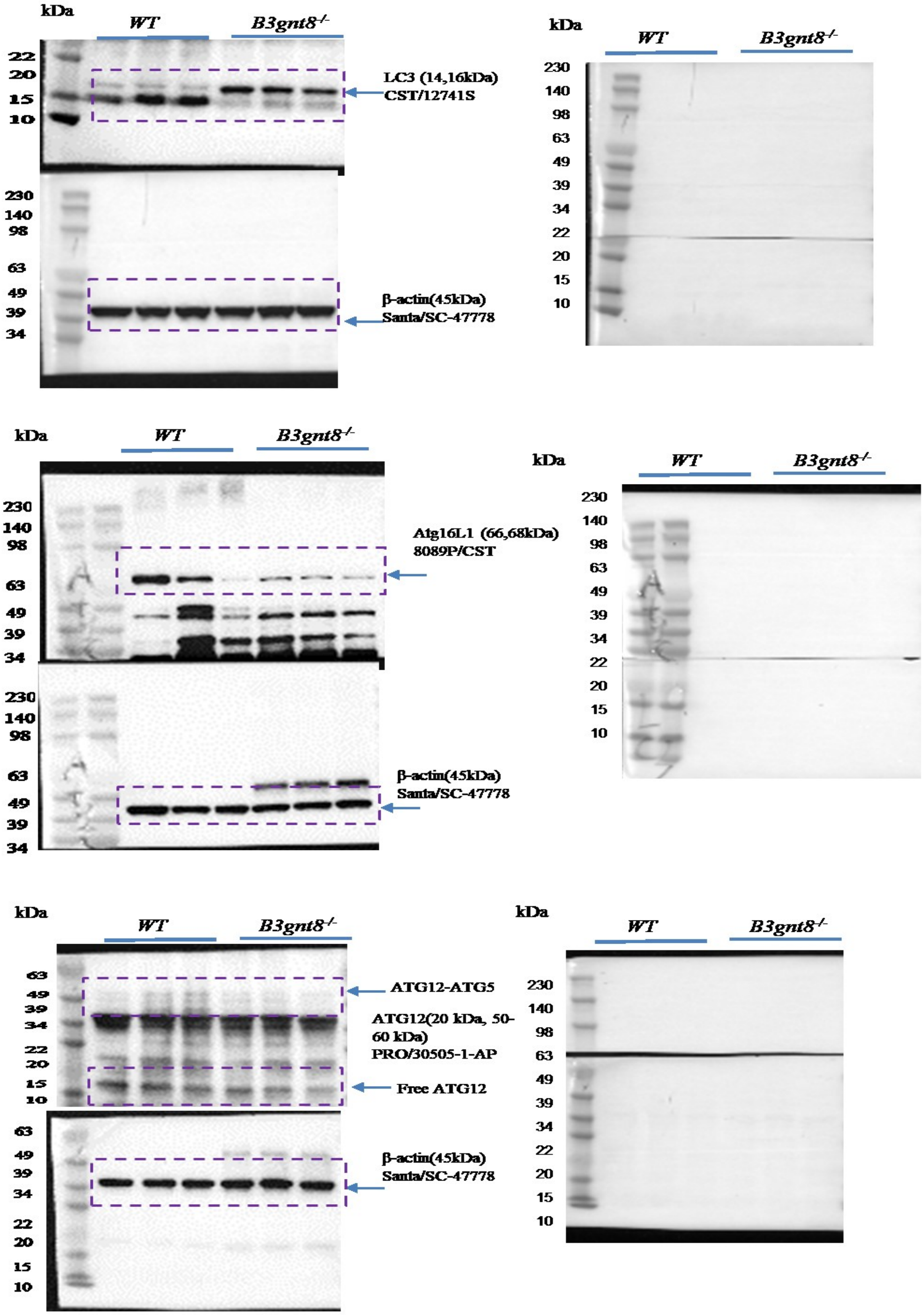
kDa
WT
B3gnt8-/-
LC3 (14,16kDa)
CST/12741S
β-actin(45kDa)
Santa/SC-47778
Atg16L1 (66,68kDa)
8089P/CST
β-actin(45kDa)
Santa/SC-47778
ATG12-ATG5
ATG12(20 kDa, 50-60 kDa)
PRO/30505-1-AP
Free ATG12
β-actin(45kDa)
Santa/SC-47778

Figure 11

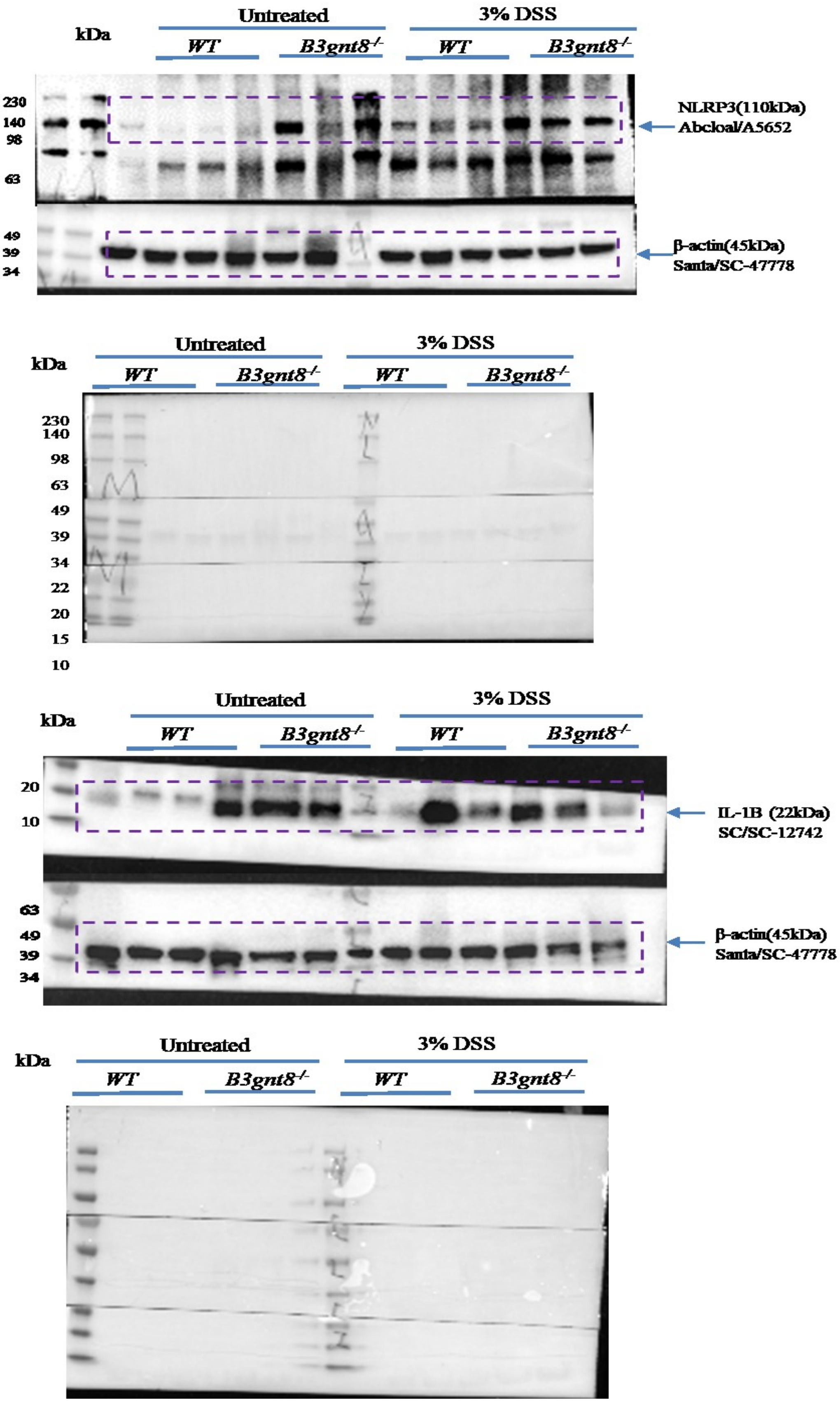

kDa
Untreated
3% DSS
WT
B3gnt8-/-
230
140
98
63
49
39
34
NLRP3(110kDa)
Abcloal/A5652
β-actin(45kDa)
Santa/SC-47778
22
20
15
10
IL-1B (22kDa)
SC/SC-12742

Figure 11

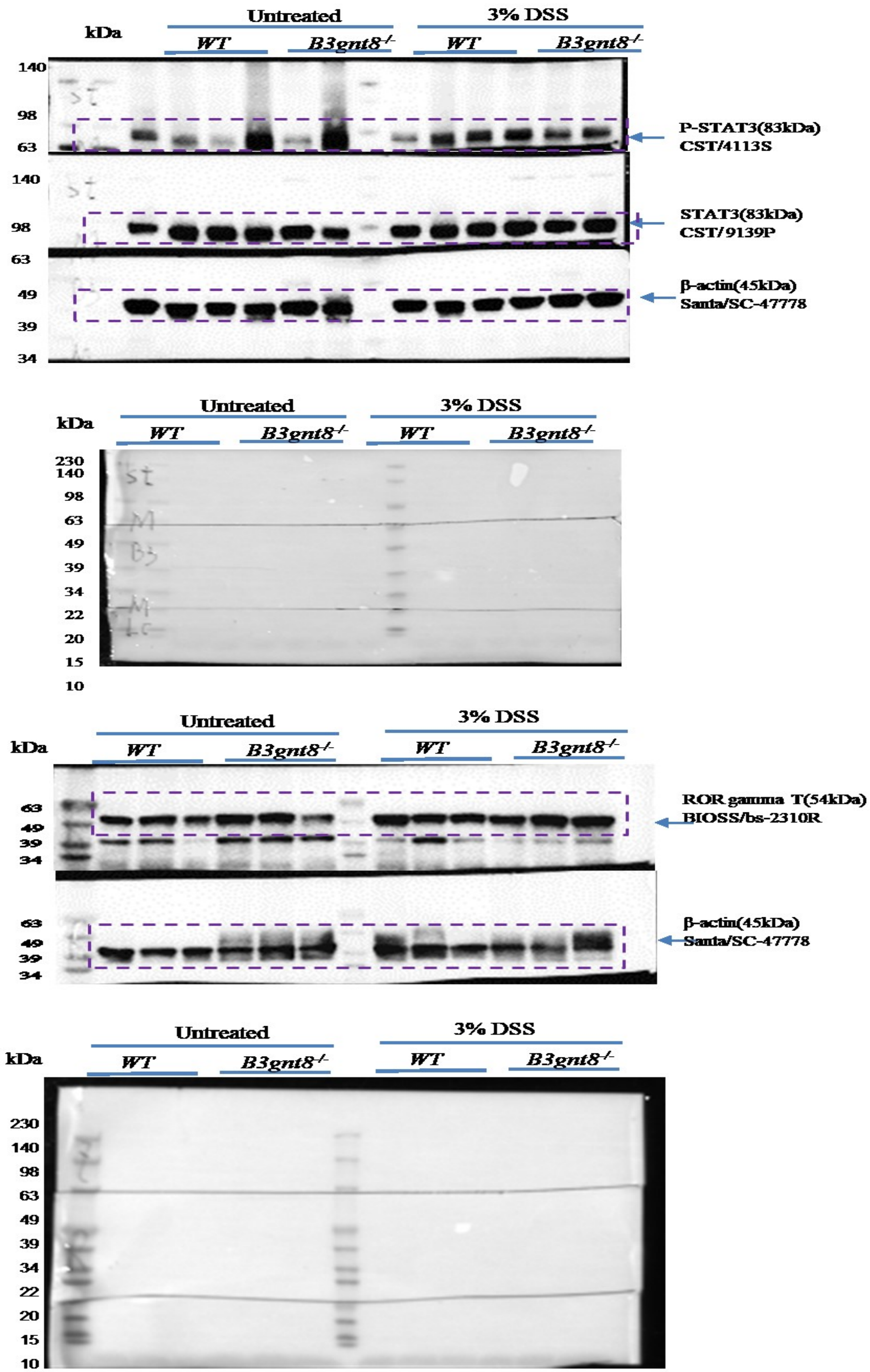
Untreated
3% DSS
kDa
WT
B3gnt8-/-
WT
B3gnt8-/-
140
98
63
140
98
63
49
39
34
P-STAT3(83kDa)
CST/4113S
STAT3(83kDa)
CST/9139P
β-actin(45kDa)
Santa/SC-47778
Untreated
3% DSS
kDa
WT
B3gnt8-/-
WT
B3gnt8-/-
230
140
98
63
49
39
34
22
20
15
10
Untreated
3% DSS
kDa
WT
B3gnt8-/-
WT
B3gnt8-/-
63
49
39
34
ROR gamma T(54kDa)
BIOSS/bs-2310R
63
49
39
34
β-actin(45kDa)
Santa/SC-47778
Untreated
3% DSS
kDa
WT
B3gnt8-/-
WT
B3gnt8-/-
230
140
98
63
49
39
34
22
20
15
10

Supplementary Figure 12 Original Immunofluorescence Staining in Figures

Figure 3A

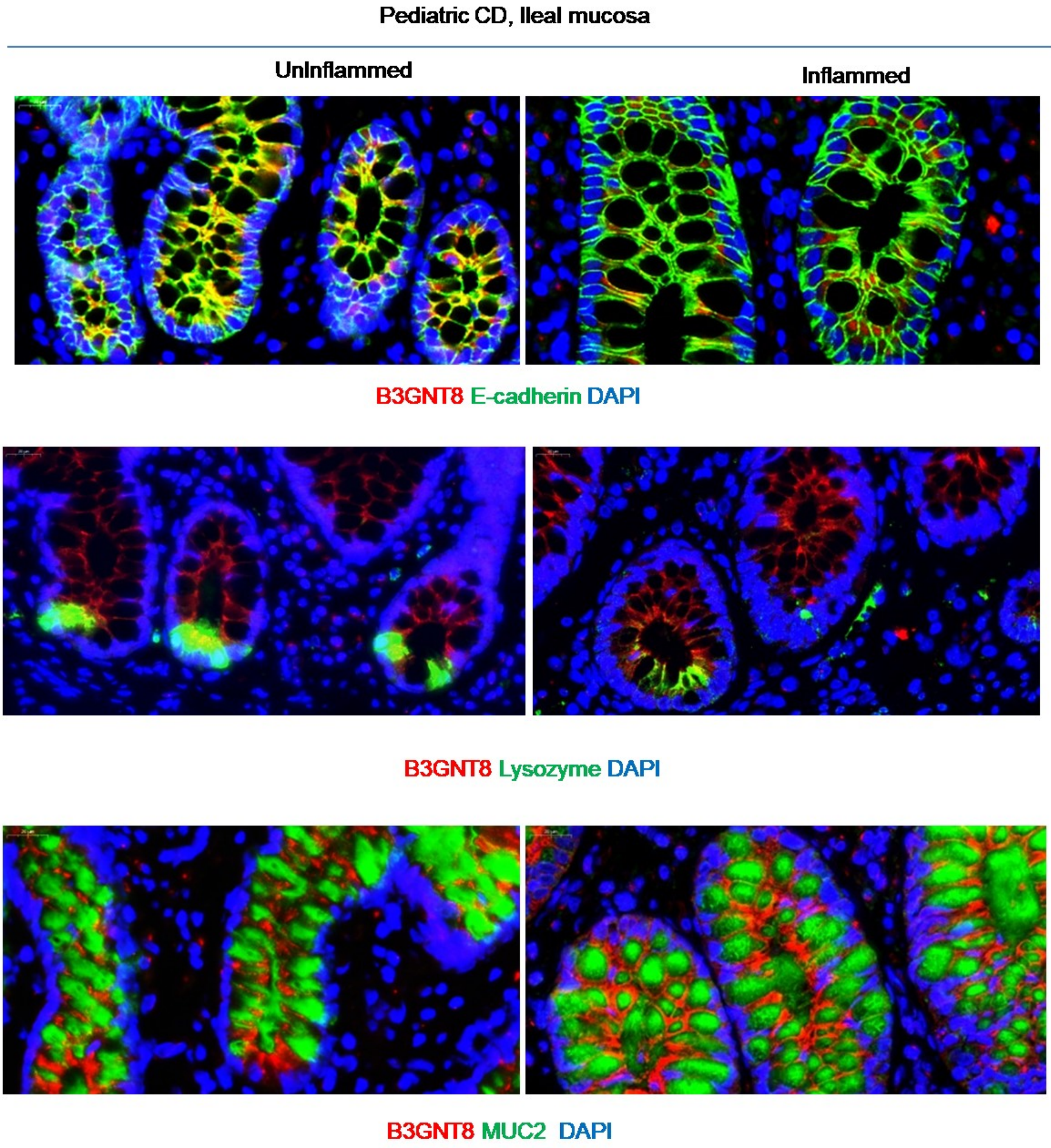

Figure 3C

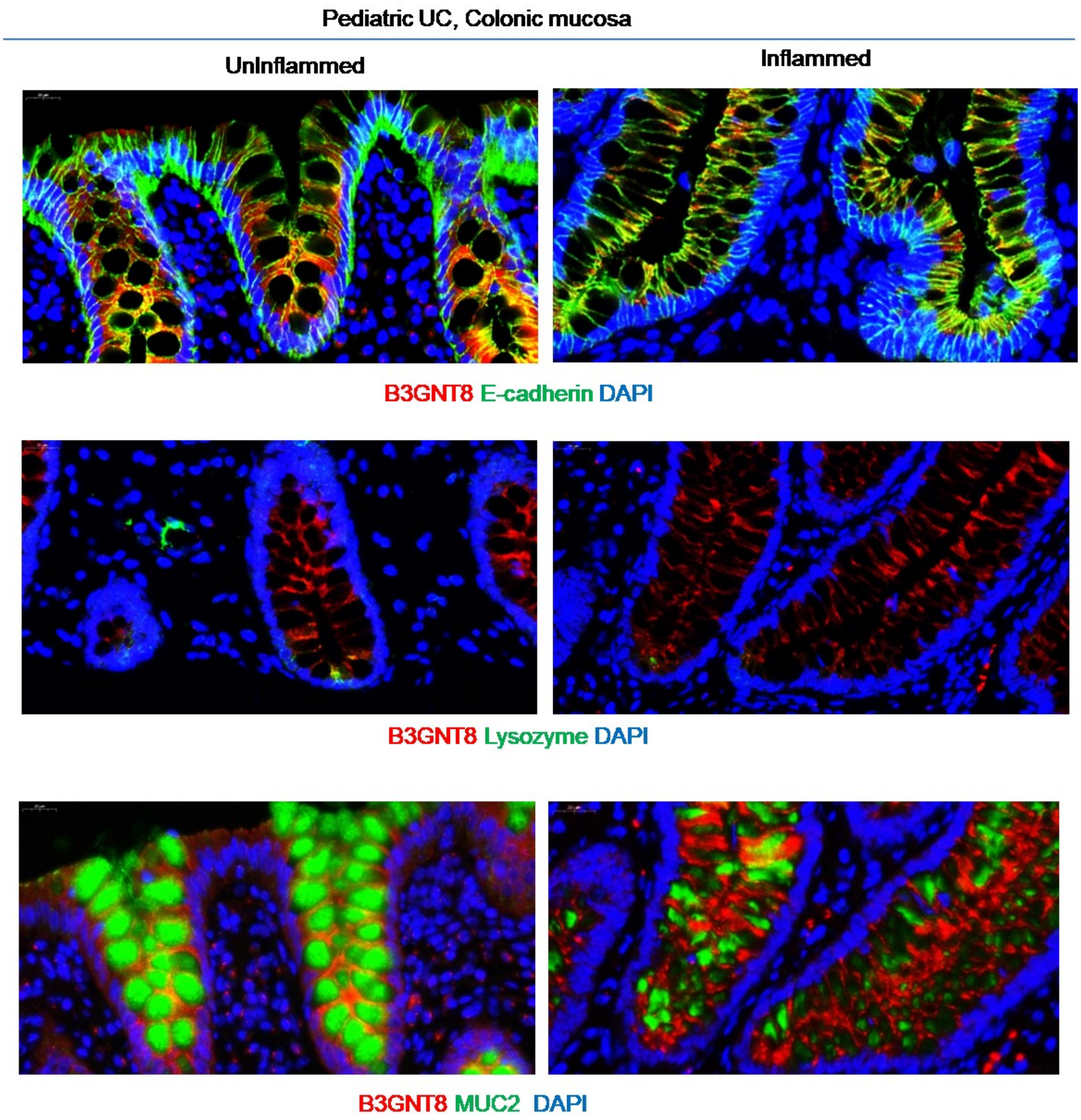
Pediatric UC, Colonic mucosa
UnInflammed
Inflammed
B3GNT8 E-cadherin DAPI
B3GNT8 Lysozyme DAPI
B3GNT8 MUC2 DAPI

Figure 4D

Dis SI

WGA MUC2 DAPI

Wt *B3gnt8$^{-/-}$*

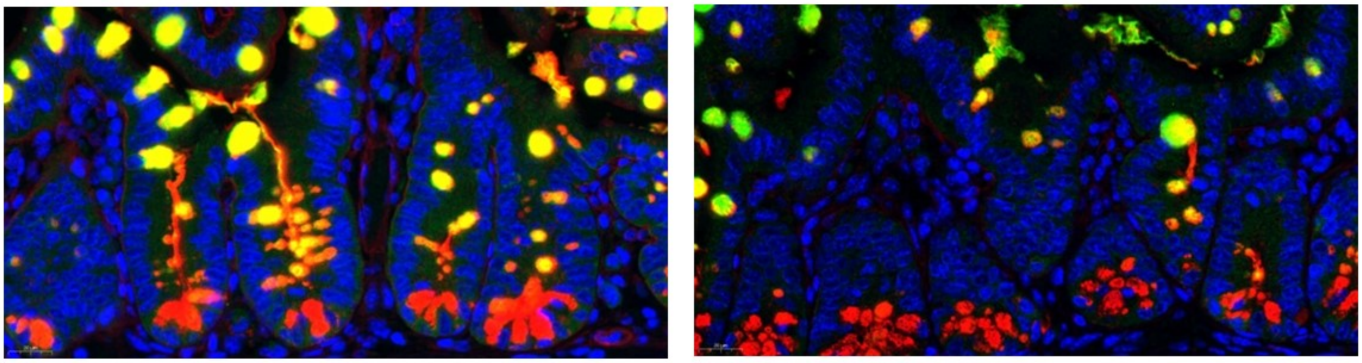

Colon

WGA MUC2 DAPI

Wt *B3gnt8$^{-/-}$*

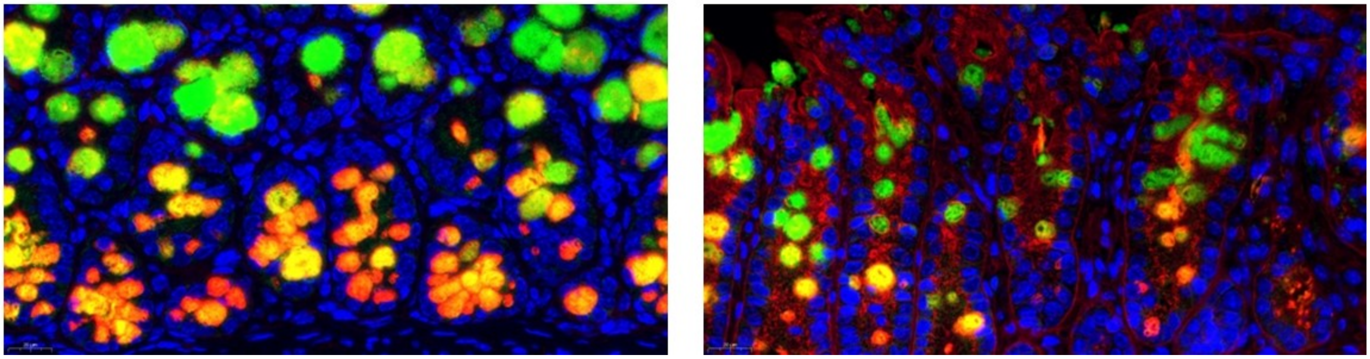

Figure 5A

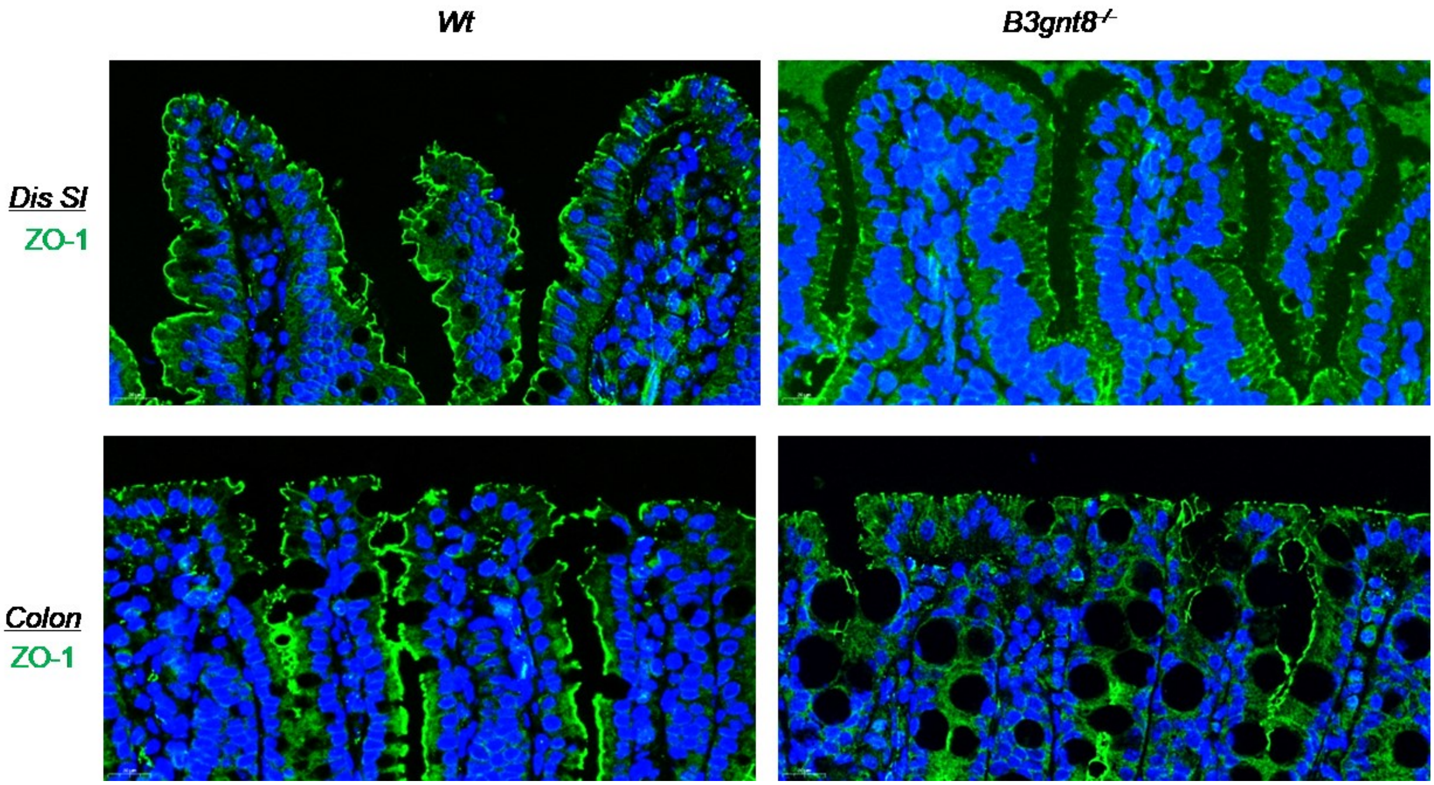

Figure 5A

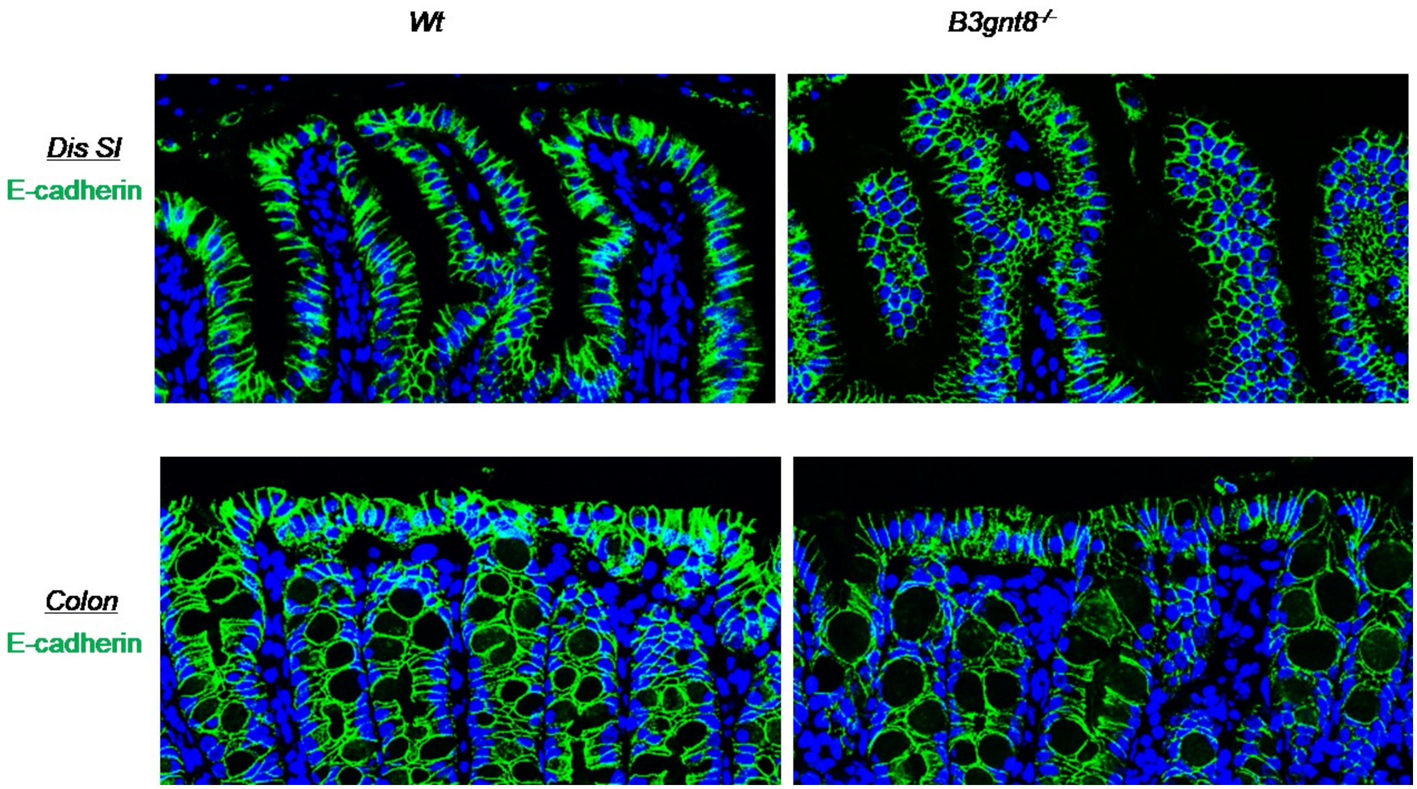

Figure 5A

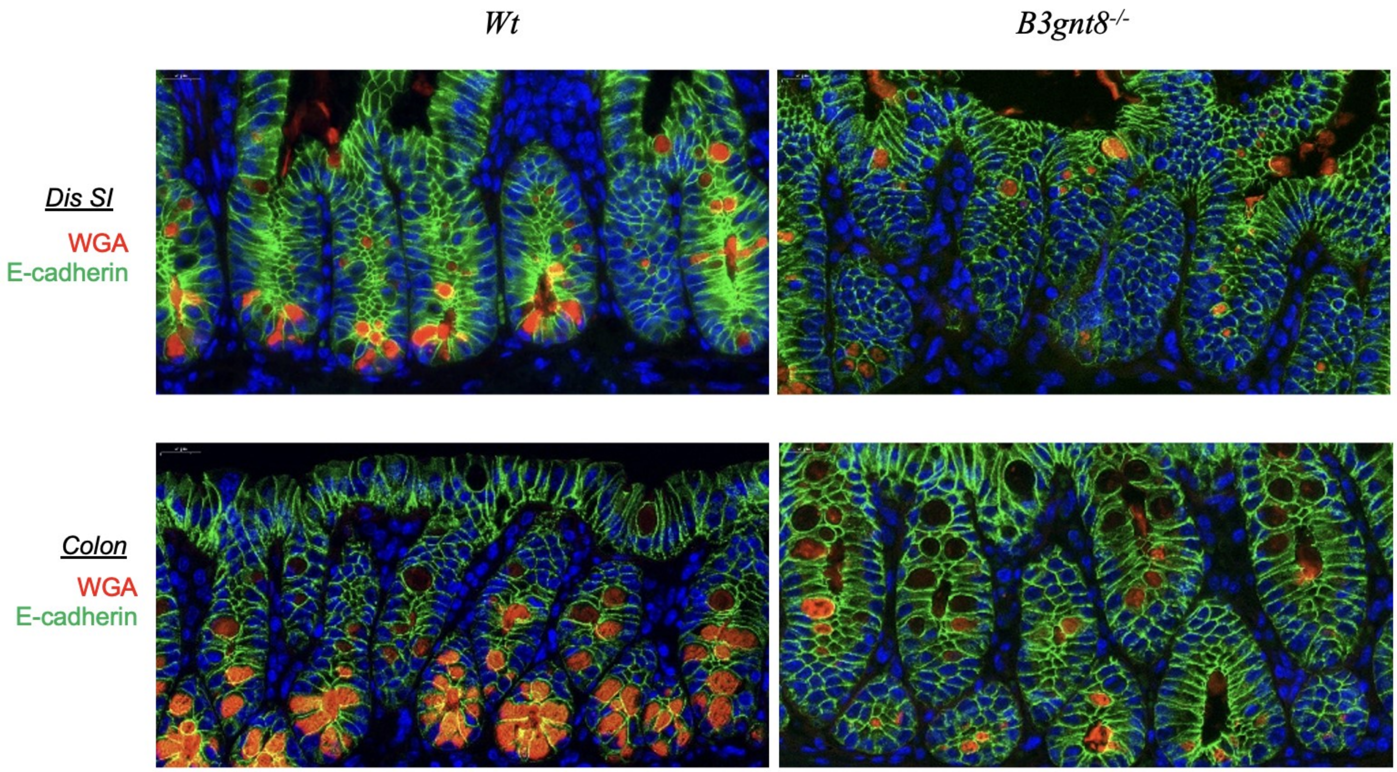

Figure 6A

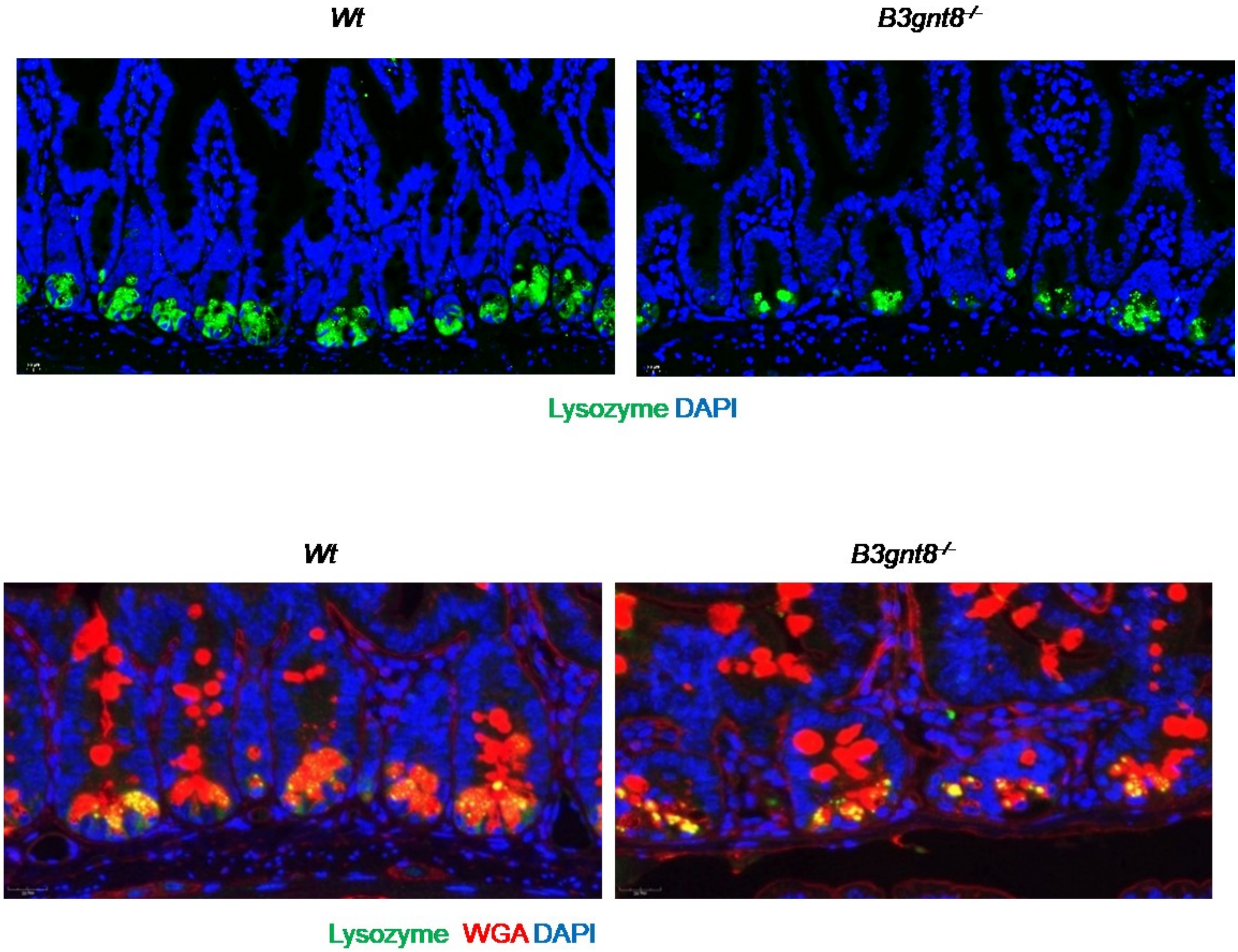

Figure 6F

Wt

LAMP2
B3GNT8
DAPI

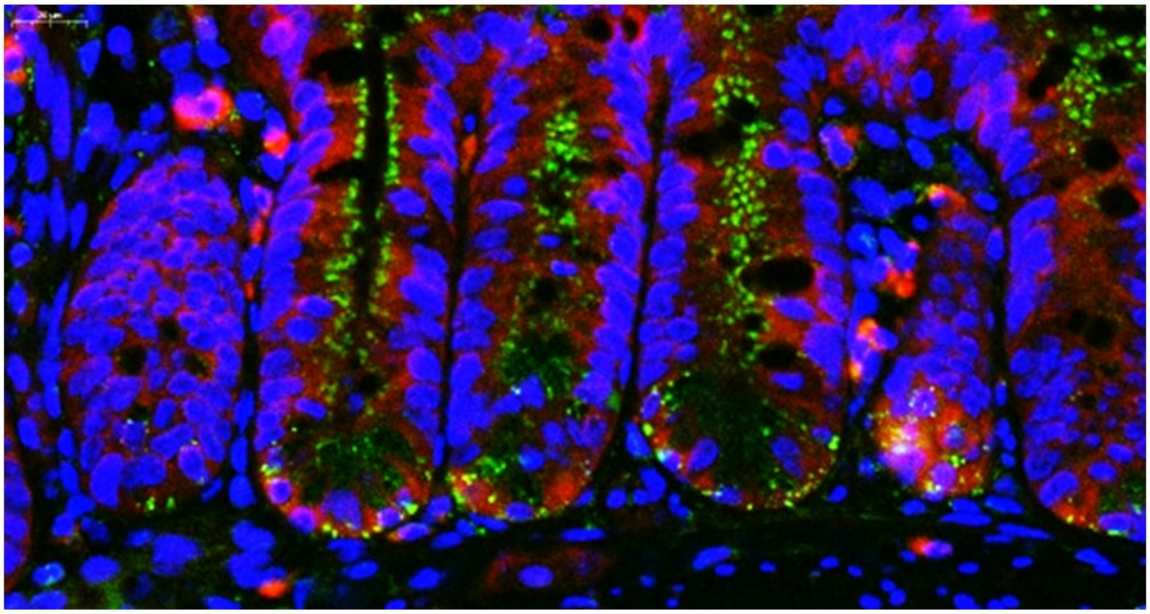

B3gnt8$^{-/-}$

LAMP2
B3GNT8
DAPI

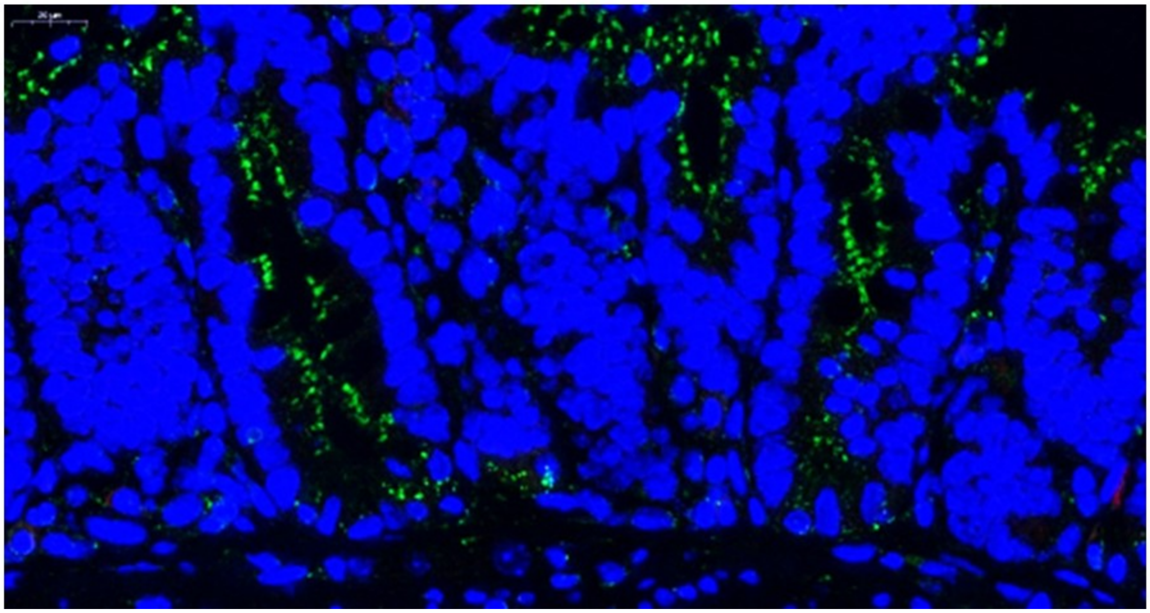

Figure 8A

Lysozyme
LC3
DAPI

Wt

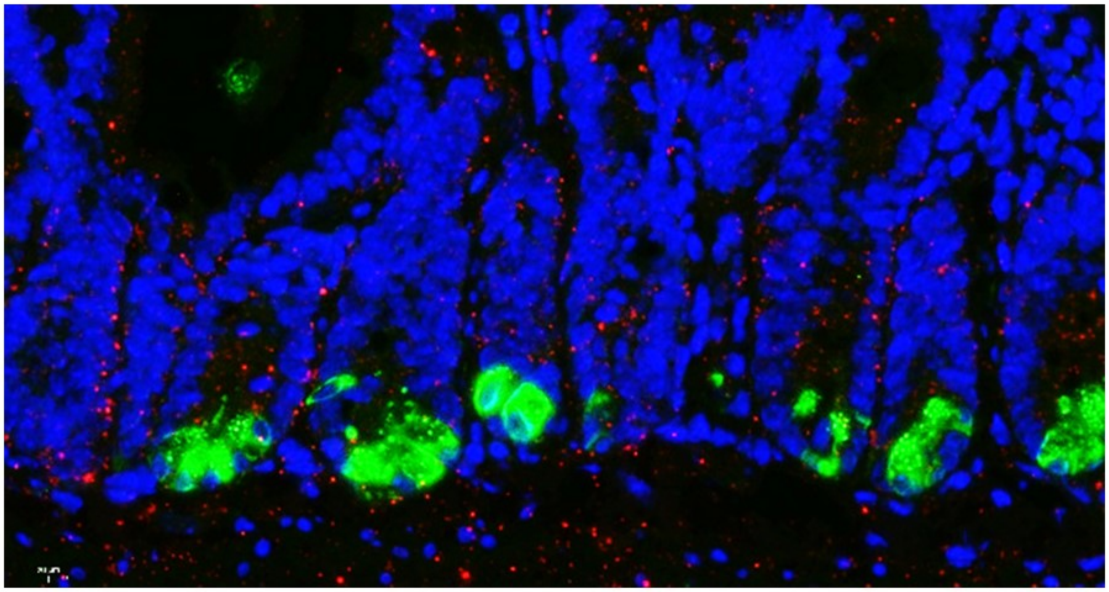

Lysozyme
LC3
DAPI

B3gnt8$^{-/-}$

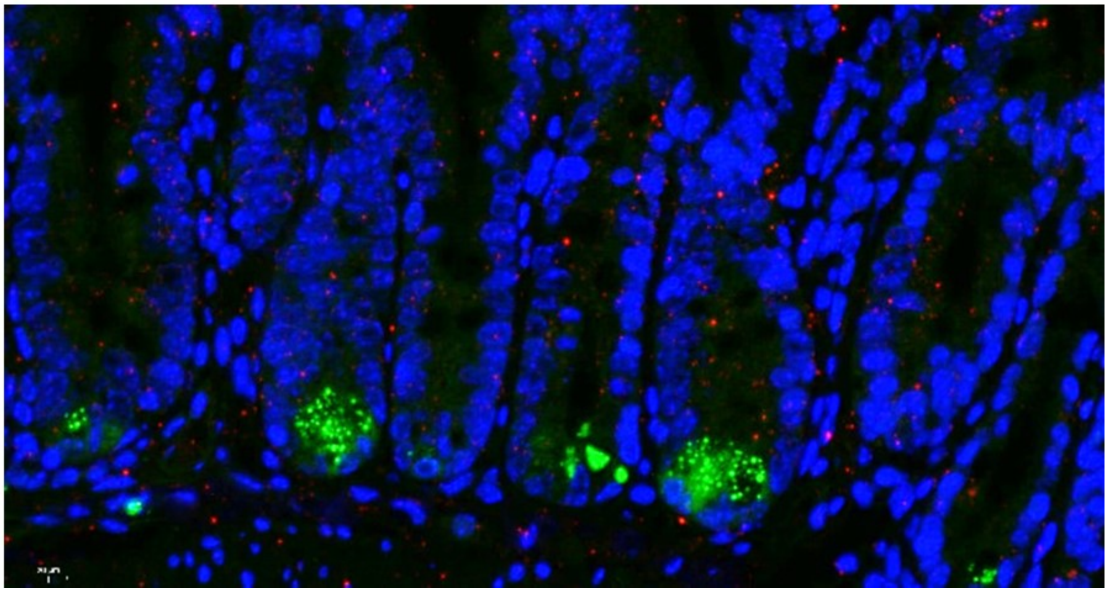

Supplementary Figure 4 A

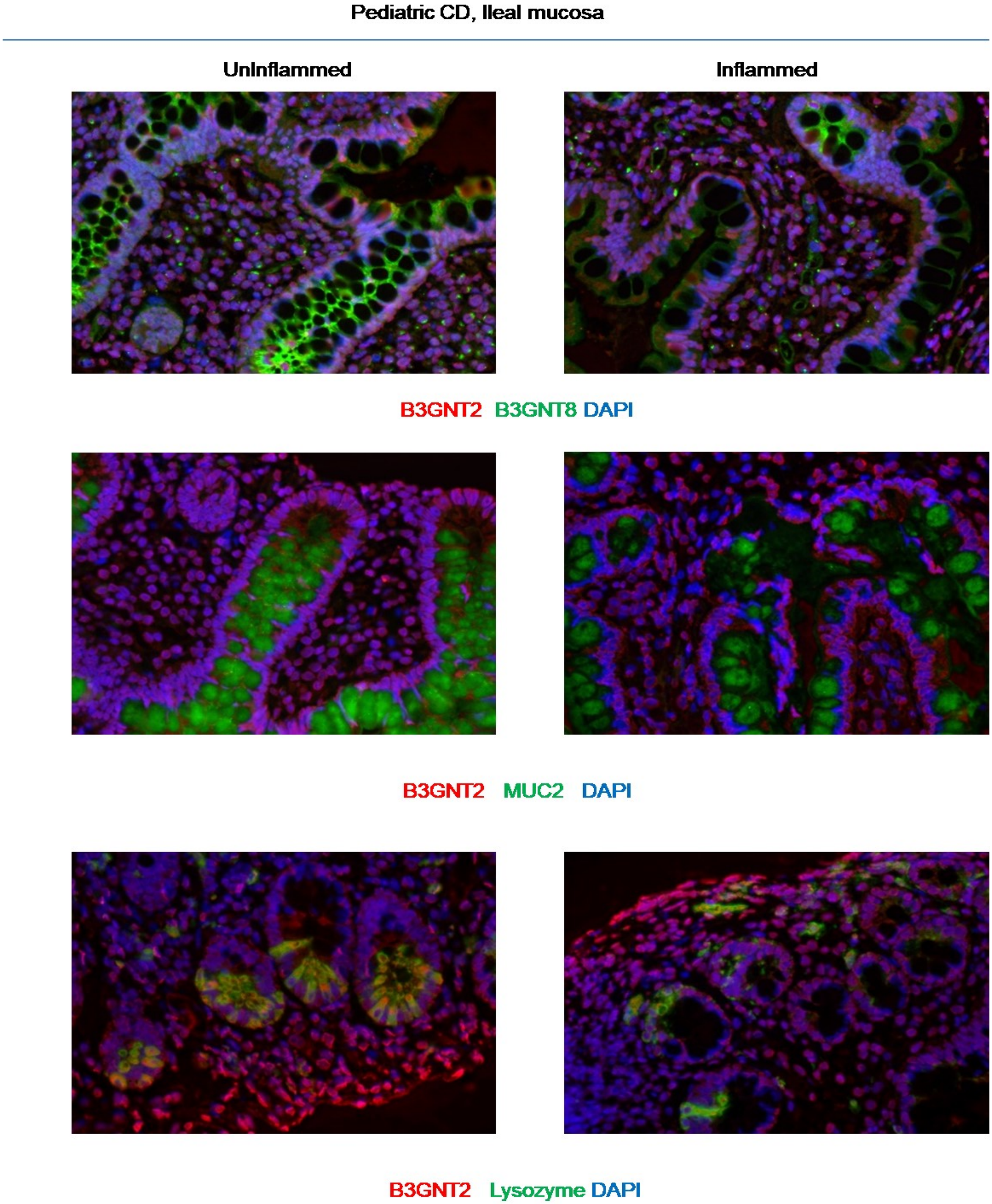

Supplementary Figure 4 B

Pediatric UC, Colonic mucosa

UnInflammed	Inflammed

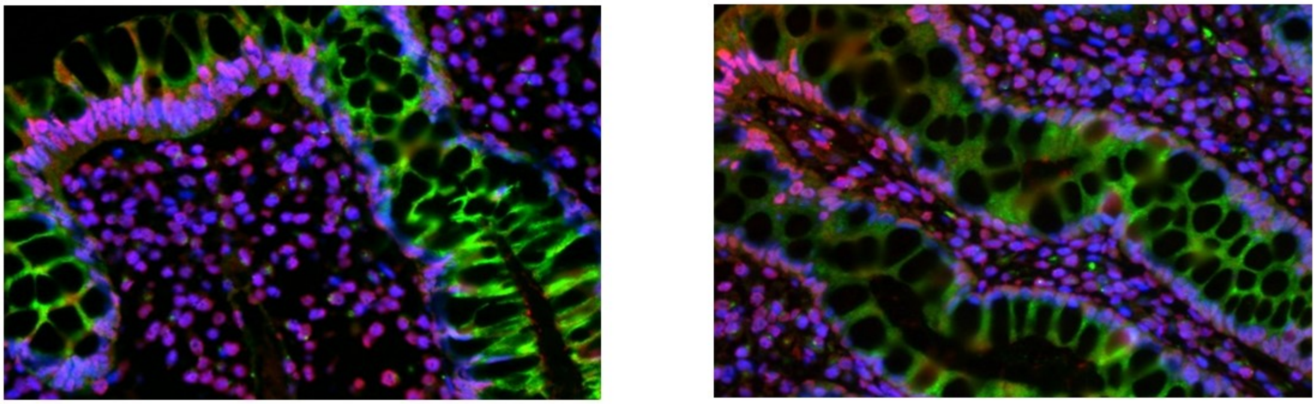

B3GNT2 B3GNT8 DAPI

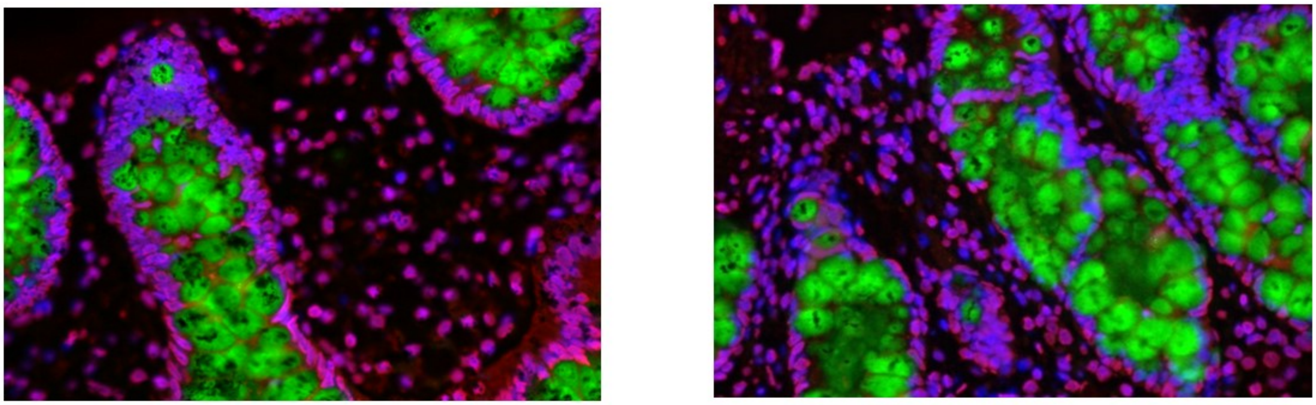

B3GNT2 MUC2 DAPI

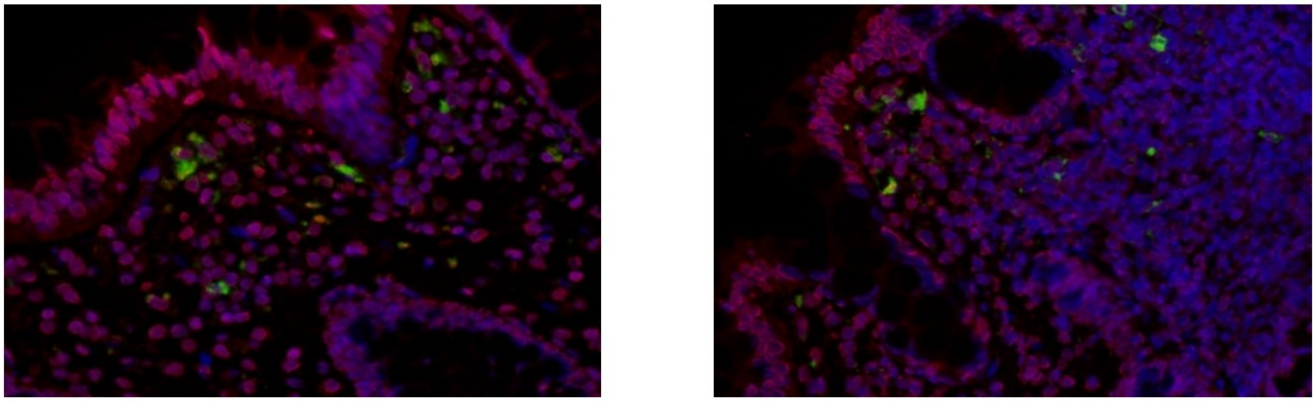

B3GNT2 Lysozyme DAPI
